# VariantMetaCaller: automated fusion of variant calling pipelines for quantitative, precision-based filtering

**DOI:** 10.1186/s12864-015-2050-y

**Published:** 2015-10-28

**Authors:** András Gézsi, Bence Bolgár, Péter Marx, Peter Sarkozy, Csaba Szalai, Péter Antal

**Affiliations:** Department of Genetics, Cell- and Immunobiology, Semmelweis University, Nagyvárad tér 4, Budapest, H-1089 Hungary; Department of Measurement and Information Systems, Budapest University of Technology and Economics, Magyar tudósok krt. 2, Budapest, H-1117 Hungary

**Keywords:** Next-generation sequencing, Variant calling, Support Vector Machine

## Abstract

**Background:**

The low concordance between different variant calling methods still poses a challenge for the wide-spread application of next-generation sequencing in research and clinical practice. A wide range of variant annotations can be used for filtering call sets in order to improve the precision of the variant calls, but the choice of the appropriate filtering thresholds is not straightforward. Variant quality score recalibration provides an alternative solution to hard filtering, but it requires large-scale, genomic data.

**Results:**

We evaluated germline variant calling pipelines based on BWA and Bowtie 2 aligners in combination with GATK UnifiedGenotyper, GATK HaplotypeCaller, FreeBayes and SAMtools variant callers, using simulated and real benchmark sequencing data (NA12878 with Illumina Platinum Genomes). We argue that these pipelines are not merely discordant, but they extract complementary useful information.

We introduce VariantMetaCaller to test the hypothesis that the automated fusion of measurement related information allows better performance than the recommended hard-filtering settings or recalibration and the fusion of the individual call sets without using annotations. VariantMetaCaller uses Support Vector Machines to combine multiple information sources generated by variant calling pipelines and estimates probabilities of variants.

This novel method had significantly higher sensitivity and precision than the individual variant callers in all target region sizes, ranging from a few hundred kilobases to whole exomes. We also demonstrated that VariantMetaCaller supports a quantitative, precision based filtering of variants under wider conditions. Specifically, the computed probabilities of the variants can be used to order the variants, and for a given threshold, probabilities can be used to estimate precision. Precision then can be directly translated to the number of true called variants, or equivalently, to the number of false calls, which allows finding problem-specific balance between sensitivity and precision.

**Conclusions:**

VariantMetaCaller can be applied to small target regions and whole exomes as well, and it can be used in cases of organisms for which highly accurate variant call sets are not yet available, therefore it can be a viable alternative to hard filtering in cases where variant quality score recalibration cannot be used. VariantMetaCaller is freely available at http://bioinformatics.mit.bme.hu/VariantMetaCaller.

**Electronic supplementary material:**

The online version of this article (doi:10.1186/s12864-015-2050-y) contains supplementary material, which is available to authorized users.

## Background

The level of uncertainty in next-generation sequencing (NGS) measurements is still higher than what is required for routine clinical use, even for germline variants in targeted gene panels and exome sequencing [1]. The measurement process includes a complex computational variant calling pipeline, which contains many alternative elements with various parameters, heavily influencing the unique characteristics and performance of the whole procedure. Several studies showed that (1) currently there is no single best general individual variant calling method with both superior sensitivity and precision at all circumstances [1, 2], and (2) there are significant discrepancies between commonly used variant calling pipelines, even when applied to the same set of sequence data [1, 3–5]. An *ad hoc* approach is the fine-tuning of the pipeline for the actual measurement, which requires substantial expertise and time, also hindering standardization and benchmarking.

Generally, variant callers aim to be sensitive, call variants “aggressively” and provide annotations to the user that can help distinguish true variants from false calls originating from sequencing, alignment or data processing artefacts. To further improve the sensitivity of the pipeline, one can use multiple variant calling methods, as it is a well-known fact that different callers produce different results [1, 3–7]. The rationale behind this practice is that the consequence of a false negative variant call (i.e. not discovering a true variant) is usually more serious than the consequence of a false positive (i.e. unreal variant claimed to be real), especially in clinical settings. The union of different call sets (called by different variant callers) could be taken for maximum sensitivity. However, this would result in higher false positive rate, i.e. a decrease in precision. Variants could, in principle, be validated experimentally using complementary measurement methods, but only at the cost of losing the high-throughput efficiency of NGS. Therefore, an application-specific balance between sensitivity and precision is needed.

A possible solution for selecting the appropriate list of variants is the use of *hard filters*. Variant callers produce a rich set of annotations that provide abundant information about mapping quality and various biases. For example, the evidence for a mutation is usually stronger at higher read depths [5]. A bias in the position of the variant in the read or a bias in the number of reads or base quality scores supporting an alternate allele may denote mapping problems and can be used to identify false variants. However, annotations have complex interrelationships [3, 5], they depend on the experimental settings, and in most cases, are difficult to interpret [2]. It is often unclear what an adequate hard filter is; beyond general guidelines each specific study requires experimenting and empirical testing. Besides, most annotation classes depend on the actual read depth, and a filter setting which works for low coverage may not perform equally well for high coverage. The non-uniform coverage often seen in NGS studies [8] makes hard filtering a challenging task. Furthermore, it is also difficult to assess the resulting precision of the hard-filtered variant set.

An automated approach to improve precision of variant calling, applicable at a larger scale, is the use of variant quality score recalibration (VQSR) [9], which can be used to reclassify variant qualities. However, it requires a large amount of data: it can be used only for whole genomes or for at least 30 whole exomes according to GATK Best Practices. If a smaller region is sequenced, one can rely only on manual hard filters. Besides, VQSR uses gold standard, “error-free” variant sets as reference. In case of organisms for which these resources are unavailable, VQSR cannot be used in a straightforward manner.

In fact, automated recalibration can be also applied using abundant annotations of multiple pipelines instead of large amount of data: in this case the heterogeneous, intermediate annotations from multiple methods can be exploited for automated “recalibration”. Indeed, this forms our central hypothesis that popular variant calling pipelines are not merely discordant, but the generated intermediate annotations contain complementary high-dimensional information, which can be combined into a better performing overall model. Our further hypothesis is that fusion of the intermediate annotation information allows the prediction of probabilities of variants in areas not accessible by current approaches.

**Fig. 1:**
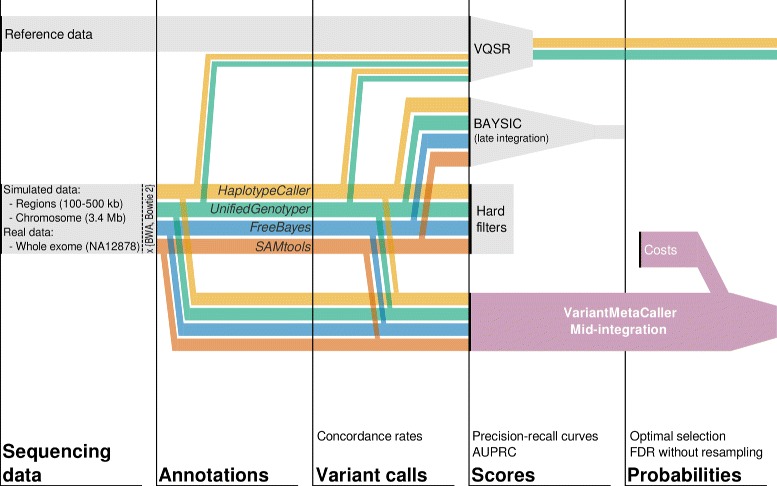
Earlier approaches, current study design including data sets and evaluations, and the conceptual overview of VariantMetaCaller. *Study design*: Simulated sequences of various target region sizes, and real sequence data covering the whole exome of NA12878 were aligned by BWA and Bowtie 2 to the human genome. Variants were called by GATK HaplotypeCaller, GATK UnifiedGenotyper, FreeBayes and SAMtools. *Evaluation*: Variant calling pipelines were compared by calculating concordance rates. Precision-recall curves were plotted and the area under the precision-recall curves was calculated for each method. *Earlier approaches*: Hard filters can be applied to filter variants by specifying annotation cutoffs. VQSR can be applied to recalibrate variant qualities based on gold standard reference data and variant annotations. BAYSIC combines the unfiltered variant calls by late integration. *Overview of VariantMetaCaller*: VariantMetaCaller (1) combines the unfiltered call sets by SVMs that use variant annotations as features and (2) estimates the probability of each variants being real. The probabilistic output of VQSR and VariantMetaCaller can be used to estimate FDR at each probability cutoff and to optimally select the filtered variants with respect to the cost function of the researchers. AUPRC = Area under the precision-recall curve, FDR = false discovery rate, NGS = Next-generation sequencing, SVM = Support Vector Machine

Based on these assumptions, we constructed VariantMetaCaller, which combines information from various variant callers using Support Vector Machines (SVM) (for an earlier related method, see [10]). Figure 1 shows the earlier approaches, the current study design including data sets and evaluations, and the conceptual overview of VariantMetaCaller. This novel method predicts the probability that a variant is a true genetic variant and not a sequencing artefact, which provides a principled solution for quantitative support for variant filtering. Specifically, probabilities can be used to order the variants, and for a given threshold, probabilities can be used to estimate precision. Precision then can be directly translated to the number of true called variants, or equivalently to the number of false calls, which allows finding problem-specific balance between sensitivity and precision, i.e. it allows a quantitative, *precision-based filtering*.

Automated fusion of multiple variant callers has been seen as a promising direction to exploit hidden information with more advanced statistical models. Until now, the arising problem of high-dimensionality and heterogeneity has remained unsolved in earlier fusion approaches, for example BAYSIC [11], used only the predicted calls, implementing late information fusion. To cope with high-dimensionality, a few SVM-based methods have already been introduced, such as the unpublished Ensemble method [12] and the one used for the Exome Sequencing Project [13]. The method of the Exome Sequencing Project was not developed to utilize the combination of multiple variant-callers, and it determines annotation value cutoffs for defining negative training examples and gold standard data sets for defining positive training examples [14]. VariantMetaCaller is conceptually similar to Ensemble, but the latter is limited to single-sample variant sets, and as to our knowledge, does not produce a quantitative score and therefore cannot be used to balance between sensitivity and precision.

In this paper, we first overview the main characteristics of synthetic data sets used for evaluation throughout the paper. Second, we report the performance of various variant calling methods with special emphasis on their heterogeneity and concordance. Next, we present a systematic evaluation and comparison of selected previous variant calling pipelines against VariantMetaCaller using both synthetic and real sequencing data. Specifically, we investigate the accuracy of the predicted probabilities of the variants showing the superiority of VariantMetaCaller over existing solutions.

## Results and discussion

### Results on simulated sequencing data

We created synthetic sequencing data with known variations in the reference genome to compare the performance of previous variant calling pipelines to that of our method. We chose chromosome 17 for illustrative purposes and created artificial diploid chromosomes that contained randomly selected exonic variants from the publicly available Exome Aggregation Consortium variant call set (see Methods). The target regions were all exons of the 17th chromosome with a total size of around 3.47 Mbp. We created 50 independent samples and arranged them into five distinct groups. The total number of SNPs and indels was 14,384 and 1,852, respectively, and the average number (and standard deviation) of polymorphic SNPs and indels was 3,132 (29.5) and 455 (12.9), respectively in the generated samples. We simulated paired-end sequencing of the artificial chromosomes at various depths of coverage from very low (4×) to high (200×) mean coverage. The simulated sequences contained Illumina-specific sequencing errors (see Methods).

After aligning the sequencing reads with BWA–MEM and Bowtie 2 to the human reference sequence, we called variants on the five sample batches with four different variant callers (GATK HaplotypeCaller, GATK UnifiedGenotyper, FreeBayes and SAMtools) (see Methods).

#### Performance and concordance of individual variant callers

Our results showed, in agreement with previous findings [2–4, 6], that there was not a single best general individual variant calling method with superior sensitivity and precision at all read depths, neither for SNPs nor indels, although HaplotypeCaller performed quite well in case of indels and was the most precise in case of SNPs (see Additional file 1: Supplementary results).

Additionally, several studies showed that there were significant discrepancies between commonly used variant calling pipelines, even when applied to the same set of sequence data [1, 3–5]. To understand and utilize this phenomenon, we systematically evaluated the concordance rates of the four variant callers, with a special focus on the impact of coverage depth. This step is essential, because our newly developed method, VariantMetaCaller, is heavily based on the concordance and certain complementarity of the unfiltered call sets of the individual variant callers.

**Fig. 2:**
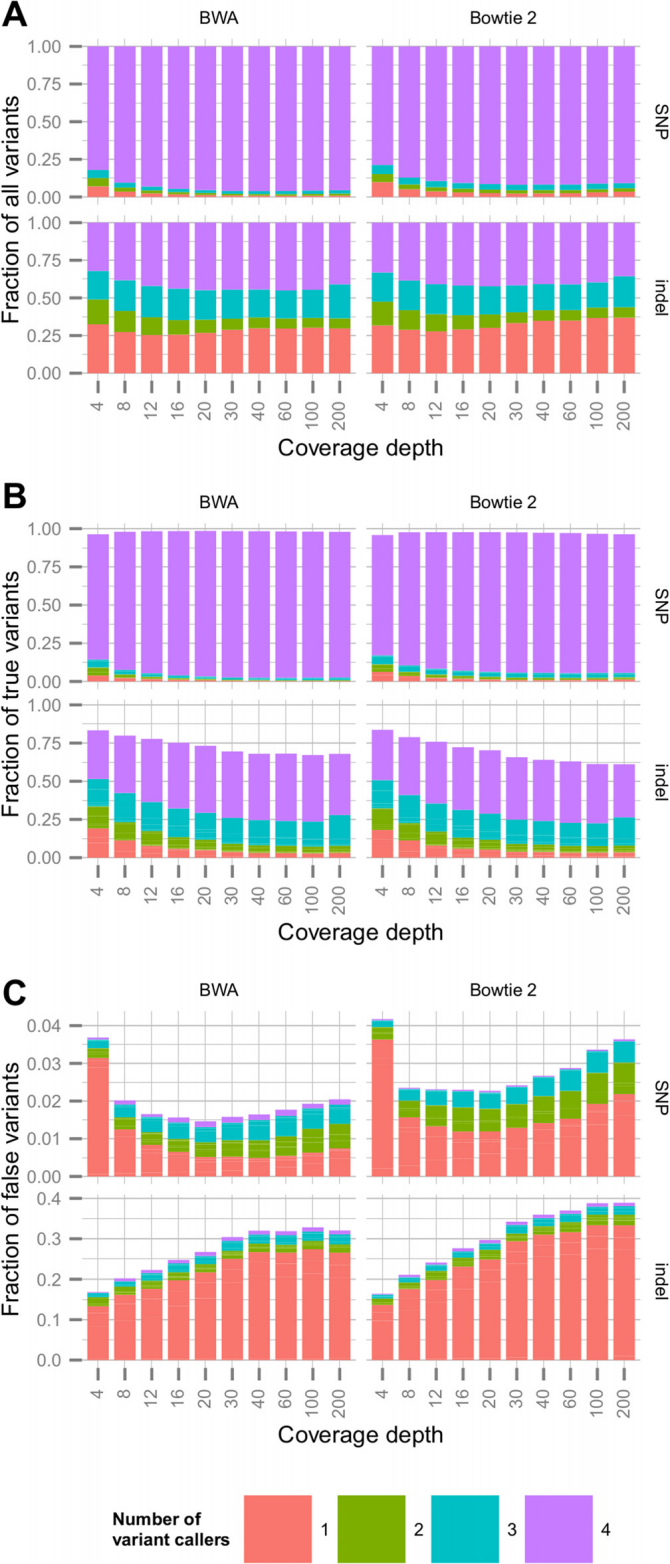
Fraction of all, true and false variants called by a different number of variant callers in case of simulated data. Sequencing reads covering the exonic region of a selected chromosome were simulated for 50 artificially generated samples with pre-known variations to the human genome (i.e. reference variants). Variants were called on the BWA–MEM and Bowtie 2 aligned reads by HaplotypeCaller, UnifiedGenotyper, FreeBayes and SAMtools. Stacked bars with different colors represent the fraction of all (**a**), true (**b**) and false (**c**) variants with respect to the reference variants, called by a given number of variant callers at various coverage depths (see the common legend on the bottom). Each panel is divided into four subpanels, where the top pair represents: SNPs, bottom pair: indels, left column: BWA alignment, right-column: Bowtie 2 alignment

First, we quantified the concordance rates of the individual variant callers by counting the number of methods calling a given variant. The percentage of concordantly called variants by all four variant callers were considerably higher for SNPs than for indels (Fig. 2). In case of SNPs, the percentage of concordant variant calls roughly increased from approximately 78−80 *%* seen in low coverage to 90−95 *%* in high coverage, depending on the aligner. Conversely, the percentage of singly-called variants roughly decreased with increasing coverage, from approximately 7−10 *%* in low coverage to 1−2 *%* in high coverage (Fig. 2a). At low depths, the frequency of the singly-called variants was the second highest, but with increasing coverage, this category became the least frequent.

In case of indels, the variant callers produced markedly different results. Irrespective of the coverage depth, less than the half of the indels were called by all four methods, and the fraction of singly-called variants were above 25 *%*. Furthermore, the frequency of the singly-called variants was the second highest at all depths.

We also found that above medium coverages, the percentage of fully concordant variants slightly began to drop for both SNPs and indels. These results are in compliance with the findings of Yu and Sun [3] and O’Rawe et al. [1], but it contradicts the expectation that with increasing read depth the accuracy of variant calling would also increase, which would in turn result in higher concordance between individual variant callers. Although, this is not the main focus of the paper, we show in the supplementary results (see Additional file 1) that sensitivity and precision change in opposite directions at varying depths. Specifically, for increasing coverage from low to medium depths, sensitivity gain surpassed precision loss alluding to increased accuracy, which also resulted in higher concordance. However, at higher read depths, the sensitivity gain and precision loss was more balanced or even reversed. We suspect that these phenomena are related to different types of statistical errors stemming from small coverage (sample size) and asymptotic errors (biases) of the variant callers, but this requires further investigation.

The concordance rates were generally lower for variant call sets based on the Bowtie 2 with respect to BWA, alignments, which can be partly explained by the relatively higher accuracy of variant calls based on BWA alignments (for the effect of the aligners on variant calling, see Additional file 1: Supplementary results).

Next, we restricted variants to only true or false positives, and partitioned the variants according to the number of methods that called them. Figure 2b and c illustrate the empirical distribution over the partitions. The fraction of true variants was generally the highest for the concordantly called SNPs and indels, and was generally the lowest for singly-called variants apart from very low coverages (Fig. 2b). In parallel with this, the fraction of falsely called variants was the highest in the category of the singly-called variants (Fig. 2c), and was negligible (<0.01 *%* for SNPs and <0.1 *%* for indels) in the case of highly concordant variants. The fraction of falsely called variants was an order of magnitude higher for indels than for SNPs, reflecting the well-known observation that indel calling is more difficult than SNP calling [1] (see Additional file 1: Supplementary results). Besides, the fraction of false variants increased with increasing coverage across all depths for indels and above 20× coverage for SNPs. At high coverage the rate of falsely called variants was approximately 2−3.6 *%* for SNPs, and 30−39 *%* for indels depending on the aligner.

Finally, we calculated the fraction of true variants among variants called by exactly one or by all four methods (Additional file 1: Figure S1). The percentage of true variants among variants called by all four methods was generally high, both for SNPs (>99.83 *%* with BWA and >99.94 *%* with Bowtie 2 alignments) and for indels (>97.6 *%*), irrespective of the coverage depth. Conversely, the fraction of true variants among singly-called variants was significantly lower, and decreased with increasing coverage, both for SNPs (with the exception of very low coverage; <50 *%* above 30× coverage) and especially for indels (<15 *%* above 30× coverage).

Our novel method, VariantMetaCaller, exploits these observations, and uses the fully concordant variants as positive and the singly-called variants as negative training examples and trains SVMs to separate true variants from potentially erroneously called variants. As Additional file 1: Figure S1 shows, there is an apparent noise in the training data, specifically the substantial fraction of true variants in the negative training set. Thus, we investigated its effects and found that excluding the negative training examples and including only the positive ones in a one-class SVM framework decreased the performance of VariantMetaCaller. Furthermore, we evaluated the performance of VariantMetaCaller using a filtered training set that was ideal in the sense that it contained only true negative singly-called and true positive fully concordant variants. However, this resulted only in a very low increase in performance (see Methods and Additional file 1: Figure S2). Finally, it can be generally expected that the use of even more variant callers in the VariantMetaCaller framework will mitigate this problem (for the effect of using only 3 callers, see Additional file 1: Table S1).

#### Comparison of VariantMetaCaller to individual variant callers

VariantMetaCaller combines the results of multiple variant callers based on their statistical properties described earlier. After merging the unfiltered variant calls, the program creates a data set for each input method from annotations generated by the callers coupled with annotations computed by VariantMetaCaller. On these data sets, SVMs are trained separately for SNPs and indels using fully concordant and singly-called variants as positive and negative training examples, respectively. A final SVM score is computed for each variant, which estimates the probability of the variant being “real” (see Methods).

**Fig. 3:**
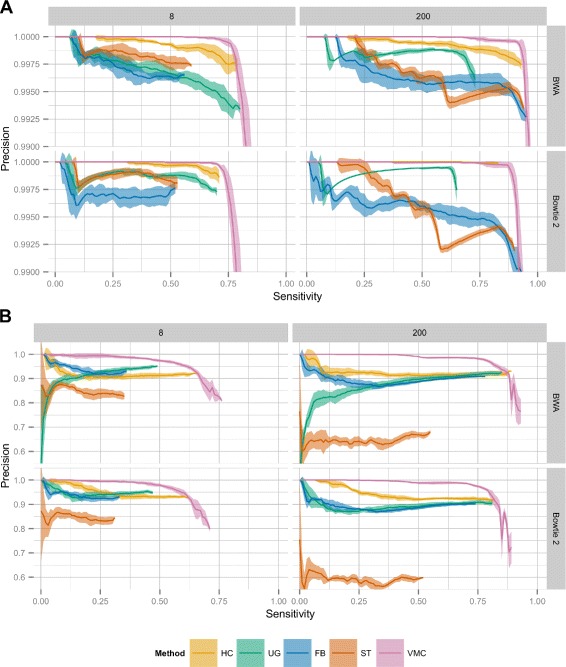
Precision-sensitivity curves at selected coverage depths for simulated data. Precision and sensitivity (recall) was calculated for each variant of the hard filtered call sets of four individual variant callers and for the results of VariantMetaCaller. Precision was plotted against sensitivity for SNPs (**a**) and for indels (**b**) at two selected coverage depths (8× and 200× representing low and high coverage, respectively). The rows differentiate between alignment algorithms and the columns represent different coverage depths. Variant calling was performed on five sample groups each containing ten samples. Bands around lines represent 95 *%* confidence intervals based on the results of the different sample groups. FB = FreeBayes, HC = HaplotypeCaller, ST = SAMtools, UG = UnifiedGenotyper, VMC = VariantMetaCaller

We evaluated the performance of VariantMetaCaller over two different pipeline sets based on the choice of the alignment software, the four variant calling methods were run on either the BWA or Bowtie 2 aligned reads. Specifically, we calculated the precision and the sensitivity (also known as recall) of all variant callers at each variant quality threshold, and similarly of VariantMetaCaller at each SVM score threshold and plotted sensitivity against precision (see Fig. 3). For the evaluations, we used the hard filtered call sets of each individual variant caller (see Methods and Additional file 1: Supplementary results). As it can be clearly seen, VariantMetaCaller dominated all variant callers in the precision–sensitivity space, meaning that VariantMetaCaller achieved higher precision at all sensitivity levels than any of the individual variant callers irrespective of the depth of coverage, the aligner and the type of the variants. Furthermore, VariantMetaCaller achieved higher maximum sensitivity as well, albeit the precision dropped sharply at high sensitivity values.

As we show in the supplementary results (see Additional file 1: Table S2), the precision of all individual variant callers was relatively high for SNPs (>0.99) as opposed to the precision for indels (0.6−0.95). The precision of VariantMetaCaller was therefore higher for SNPs than for indels.

We calculated the area under the precision–recall curves (AUPRC), which is a summary statistic that reflects the ability of a score to correctly identify true variants. More specifically, the AUPRC measure can be interpreted as a probability: it equals the fraction of true variants among those variants whose score exceed a randomly selected threshold [15]. AUPRC is commonly used to assess the performance in highly imbalanced problems, where true negatives highly outnumber the true positives, such as document search on web, and this also holds in variant calling, where the true negatives virtually span the whole target region. AUPRC is a more informative indicator of performance in these cases than e.g. the area under the receiver operating characteristic curve (AUROC), because AUPRC is not overwhelmed by the huge number of true negatives.

The AUPRC of VariantMetaCaller was the highest among all methods independently of coverage depth, the aligner or the type of the variants (Additional file 1: Figure S3, Figure S4, Table S3). The difference was strongly statistically significant in the case of all depths and for both aligners (maximum Bonferroni-adjusted p-value in case of SNPs: 0.002, indels: 0.006, paired two-tailed t-test across sample groups; Additional file 1: Table S3). As it is expected, the AUPRC was lower at low depths and increased with higher coverage, and for SNPs, the AUPRC was higher at the same depth than for indels. In case of SNPs (Additional file 1: Figure S3A), the individual variant caller with the highest AUPRC varied with different depths and aligners. At the lowest depth, UnifiedGenotyper had the highest AUPRC, but with increasing depth it became the worst. HaplotypeCaller produced the highest AUPRC for indels among the variant callers irrespective of the depth of coverage and the aligner (Additional file 1: Figure S3B).

Generally, the difference between VariantMetaCaller and HaplotypeCaller was the lowest with the exception of very low depths, and except for SNPs with Bowtie 2 alignment where the best performing method varied depending on the coverage depth. In case of SNPs (Additional file 1: Figure S4A), both FreeBayes and SAMtools showed a relatively high difference at low depths, but these differences decreased sharply at higher coverage. Interestingly, UnifiedGenotyper showed an opposite trend: relatively good performance at low depths turned into the largest difference at higher depths. In case of indels (Additional file 1: Figure S4B), the differences of AUPRC between VariantMetaCaller and HaplotypeCaller, UnifiedGenotyper and FreeBayes decreased slightly with increasing depth, and the differences between VariantMetaCaller and SAMtools increased with increasing depth.

The observed varying performance of the methods compared to VariantMetaCaller is due to many factors: (1) the relative sensitivity of the individual variant callers to each other (see Additional file 1: Figure S5), (2) the trends of the false discovery rates of the individual variant callers (see Additional file 1: Figure S6), (3) the varying sensitivity loss and precision change caused by hard filtering (see Additional file 1: Figure S7) and (4) the goodness of the variant quality estimation of the variant callers.

##### Effects of the aligner

The variant callers generally achieved higher maximum sensitivity when BWA, as opposed to Bowtie 2, was used for alignment (Additional file 1: Table S4). Consequently, VariantMetaCaller achieved higher maximum sensitivity and AUPRC when BWA was used at all read depths for both SNPs and indels (Table 1 and Additional file 1: Table S5). In case of SNPs, the mean difference between maximum sensitivity achieved by BWA and Bowtie 2 alignment across all coverage depths and sample groups was 0.028 (95 % CI: 0.027−0.029). Although the differences seem small, 0.01 *%* gain in sensitivity denotes discovering approximately 144 additional variants in the current experimental setting. In case of indels, the difference between maximum sensitivity was even larger: 0.044 (95 % CI: 0.042−0.046). In the current setting, 0.01 *%* gain in sensitivity denotes discovering approximately 19 additional indels. The overall precision and the AUPRC scores of VariantMetaCaller were also higher in case of BWA alignment than Bowtie 2 alignments for both SNPs and indels (Table 1). The AUPRC differences notably varied with the used aligner in case of SNPs (Additional file 1: Figure S4A), but showed very similar patterns in case of indels (Additional file 1: Figure S4B). For further details of the effects of the aligners, see supplementary results (see Additional file 1).

**Table 1:** Effects of the differences between BWA and Bowtie 2 alignments on the accuracy of variant calling in simulated data set

Difference between BWA and Bowtie 2 in terms of	SNPs			Indels		
	Mean difference	95 % CI	*P*-value	Mean difference	95 % CI	*P*-value
Maximum sensitivity of VariantMetaCaller	0.028	0.027−0.029	2.2×10^−16^	0.044	0.042−0.046	2.2×10^−16^
Precision at maximum sensitivity	0.009	0.007−0.01	2.2×10^−16^	0.039	0.025−0.053	8.6×10^−7^
AUPRC of VariantMetaCaller	0.028	0.027−0.029	2.2×10^−16^	0.042	0.040−0.044	2.2×10^−16^

#### Results at various sizes of genomic regions

In order to demonstrate the applicability of VariantMetaCaller on smaller genomic scales, especially in case of target region sizes that are typical in targeted gene panels, we filtered the full length chromosome to smaller non-overlapping regions, where the exonic length added up to approximately 100 kb, 200 kb, 300 kb and 500 kb, respectively. We selected ten regions for each size and performed the analyses on each region. The number of variants in the regions is shown in Table S6 (see Additional file 1).

The AUPRC of VariantMetaCaller was the highest among all methods irrespective of the size of the region, the coverage depth, the aligner and the type of the variants (Additional file 1: Figure S8). The difference was strongly statistically significant in case of all genomic sizes, depths and aligners (maximum Bonferroni-adjusted p-value in case of SNPs: 0.041, indels: 0.005, paired two-tailed t-test across sample groups and different regions of the same size). Similarly to the case of full length chromosome, the AUPRC for SNPs was higher at the same depth than for indels.

The difference between AUPRC of VariantMetaCaller and each variant caller varied mostly with the coverage depth and showed similar patterns across the different sizes of the target regions and the used aligner (Additional file 1: Figure S9). In case of SNPs, FreeBayes performed generally well, i.e. had the lowest difference of AUPRC compared to that of VariantMetaCaller (Additional file 1: Figure S9A), but in case of indels, HaplotypeCaller performed consistently better (Additional file 1: Figure S9B).

When the BWA aligner was used instead of Bowtie 2, the AUPRC of VariantMetaCaller was statistically significantly higher: the mean difference of AUPRC across all depths of coverage, sample groups and different regions was 0.032 (95 % CI: 0.03−0.035, p-value: <2.2∗10^−16^, paired two-tailed t-test) and 0.04 (95 % CI: 0.036−0.043, p-value: <2.2∗10^−16^, paired two-tailed t-test) in the case of SNPs and indels, respectively.

These results demonstrate the validity of VariantMetaCaller also in case of target regions that are typical in targeted gene panels.

### Results on real sequencing data

We also evaluated VariantMetaCaller on real sequencing data, using the publicly available data set of NA12878, for which a high confident “platinum” quality reference variant call set [16] is also available from *Illumina, Inc.*. We aligned the quality filtered sequencing reads with BWA–MEM and Bowtie 2, filtered the alignments to the whole exome, and performed base quality score recalibration and indel realignment according to the GATK Best Practices. The mean coverage depth was approximately 12× in case of both alignments. We called SNPs and indels with the four selected variant callers as before (see Methods).

The concordance of the unfiltered variant call sets called by the individual methods was modest (Table 2). The percentage of SNPs called concordantly by all four variant callers was 88.8 and 84.27 *%* for BWA and Bowtie 2 alignments, respectively. The percentage of variants that were called by only one variant caller was 3.48 *%* for BWA alignments and even higher, 8.83 *%* for Bowtie 2 alignments. The concordance rates were lower for indels: less than the half of all indels were called by all four callers, and the percentage of singly-called variants was 21.36 *%* (BWA) and 20.43 *%* (Bowtie 2).

**Table 2:** Number of unfiltered variants in the real data set called by the individual variant callers

Variant found by variant caller				SNPs				Indels			
				BWA		Bowtie 2		BWA		Bowtie 2	
HC	UG	FreeBayes	SAMtools	# of variants	% of all	# of variants	% of all	# of variants	% of all	# of variants	% of all
+	+	+	+	48297	88.81 %	43659	84.27 %	2918	46.73 %	2958	49.58 %
+	+	+		440	0.81 %	297	0.57 %	532	8.52 %	544	9.12 %
+	+		+	432	0.79 %	273	0.53 %	76	1.22 %	77	1.29 %
+		+	+	296	0.54 %	215	0.42 %	638	10.22 %	466	7.81 %
	+	+	+	1332	2.45 %	1078	2.08 %	53	0.85 %	66	1.11 %
+	+			222	0.41 %	115	0.22 %	18	0.29 %	24	0.40 %
+		+		82	0.15 %	45	0.09 %	254	4.07 %	186	3.12 %
+			+	57	0.10 %	36	0.07 %	235	3.76 %	130	2.18 %
	+	+		367	0.67 %	403	0.78 %	56	0.90 %	99	1.66 %
	+		+	164	0.30 %	90	0.17 %	23	0.37 %	41	0.69 %
		+	+	798	1.47 %	1021	1.97 %	108	1.73 %	156	2.61 %
+				499	0.92 %	178	0.34 %	578	9.26 %	342	5.73 %
	+			329	0.60 %	330	0.64 %	11	0.18 %	40	0.67 %
		+		781	1.44 %	3596	6.94 %	256	4.10 %	371	6.22 %
			+	285	0.52 %	471	0.91 %	489	7.83 %	466	7.81 %

We combined the unfiltered variant call sets of the four variant callers by VariantMetaCaller. Again, during the SVM training, we used the concordant variants (i.e. called by all four variant callers) as positive and the singly-called variants as negative training examples. After fusing the annotation data sets with SVMs, we estimated the probability of each variant being real (see Methods). We also combined the individual call sets with BAYSIC [11], which performs a latent class analysis and estimates a posterior probability for each variant. In addition, we performed GATK VQSR for the variants called by HaplotypeCaller and UnifiedGenotyper. VQSR fits a Gaussian mixture model to the quantitative annotations given to each variant and estimates a posterior probability to each variant call. In order to be able to use VQSR on the exome of a single sample, we restricted the number of the fitted Gaussians to 4 according the current recommendations [17]. Finally, we restricted all variant call sets to the confident region of the Platinum reference call set.

**Fig. 4:**
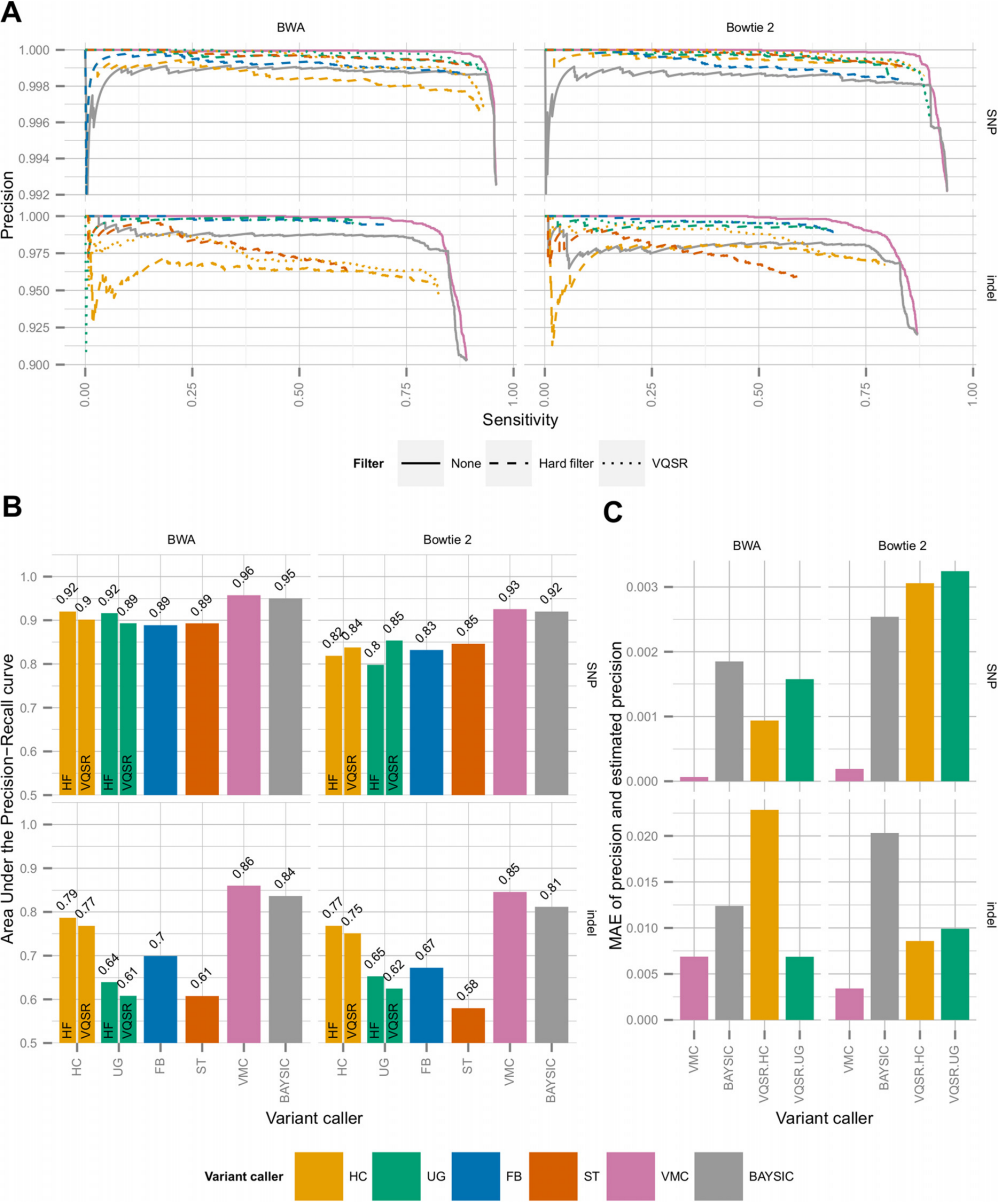
Results on real sequencing data. Sequence reads originating from a single lane of an Illumina HiSeq 2000 run of NA12878 were aligned by BWA and Bowtie 2 to the human genome and the alignments were filtered to the target region of the whole exome. Variants were called by GATK HaplotypeCaller, GATK UnifiedGenotyper, FreeBayes and SAMtools and the unfiltered call sets were combined by VariantMetaCaller and BAYSIC. Each variant call sets were hard filtered according to general recommendations and the GATK-based variant calls were also recalibrated by VQSR. All call sets were filtered to the confidently called region of the Platinum reference call set. **a** Precision-recall curves for each method for SNPs (top) and for indels (bottom) in case of BWA (left) and Bowtie 2 (right) alignment. **b** Area under the precision-recall curves for each method for SNPs (top) and for indels (bottom) in case of BWA (left) and Bowtie 2 (right) alignment. **c** Mean absolute error of estimated versus true precision of the different methods that produce probability estimates of variants. FB = FreeBayes, HC = HaplotypeCaller, HF = Hard filtered, MAE = mean absolute error, ST = SAMtools, UG = UnifiedGenotyper, VMC = VariantMetaCaller, VQSR = Variant quality score recalibration

We computed the precision and sensitivity for each hard filtered variant call set, for the variant quality score recalibrated variant sets, for BAYSIC and for VariantMetaCaller. VariantMetaCaller generally dominated all other methods in the precision–sensitivity space, meaning that VariantMetaCaller achieved higher precision at most sensitivity levels than any of the other method independently of the aligner and the type of the variants (Fig. 4a). This is also reflected by the finding that VariantMetaCaller had the highest AUPRC (Fig. 4b). The relative performance of the individual variant callers was similar to that of observed using synthetic data. In case of SNPs and BWA alignments, the hard-filtered HaplotypeCaller and UnifiedGenotyper had roughly equal AUPRCs (0.92), and higher than that of FreeBayes and SAMtools (0.89 both). However, using Bowtie 2 alignments, SAMtools performed better than any of the individual variant callers (AUPRC: 0.85), and UnifiedGenotyper proved to be the worst (AUPRC: 0.8). In case of indels the results qualitatively mirrored those seen for synthetic data (for comparison, see Fig. 4b to Additional file 1: Figure S3), except that the relative performance of UnifiedGenotyper and FreeBayes was reverted. VQSR improved performance relative to hard filtering only in the case of SNPs and Bowtie 2 alignments. This result may be related to the scarcity of data relative to the high demands of VQSR.

To test our central hypothesis about the advantage of intermediate information fusion, we compared VariantMetaCaller to the late information fusion method BAYSIC (see Fig. 4b). We found that the difference between the AUPRC of VariantMetaCaller and BAYSIC was in the range of 1−4 *%*. This is remarkable, because 1 *%* difference means prioritizing approximately 473 SNPs, and 49 indels more accurately. Additionally, we also computed the AUPRC for VariantMetaCaller and BAYSIC for each chromosome, and found that the AUPRC for VariantMetaCaller was higher than that for BAYSIC in most cases regardless of the type of the variant or the aligner and the differences were strongly statistically significant (Table 3).

**Table 3:** Differences between AUPRC of VariantMetaCaller and BAYSIC in case of each human chromosome

Type	Aligner	Nr. of chromosomes ^*a*^	*P*-value ^*b*^
SNP	BWA	21	5.96×10^−7^
SNP	Bowtie 2	22	2.15×10^−5^
Indel	BWA	19	5.33×10^−5^
Indel	Bowtie 2	21	3.93×10^−6^

^*a*^Number of chromosomes for which the AUPRC of VariantMetaCaller was greater than of BAYSIC

^*b*^Computed with one-tailed, paired Wilcoxon test

The other central theme of our work is to provide a flexible solution for quantitative support of variant filtering, similarly to the false discovery rate based paradigm [18–20]. This can be achieved by the precise estimation of the probability of the variants. Specifically, probabilities can be used to order the variants, and for a given threshold, unlike scores, probabilities can be used to estimate precision (see Eq. 2). Precision then can be directly translated to the number of true called variants, or equivalently to the number of false calls, which supports quantitative, application-specific filter adjustment. As VariantMetaCaller, BAYSIC and VQSR estimate the probability of each variant, we also investigated the differences of the methods with respect to the goodness of this estimation. For this purpose, we calculated the mean absolute error (MAE) of the predicted and the real precision for each method. The MAE rate was generally low, but it was the lowest in the case of VariantMetaCaller for SNPs irrespective of the aligner, and it was the lowest for indels with Bowtie 2 alignment (Fig. 4c). In case of indels with BWA alignment, the MAE of VariantMetaCaller was comparable to that of UnifiedGenotyper, but the latter had substantially lower sensitivity. In summary, the predicted precision or the predicted false discovery rate can be conveniently used for filtering. Furthermore, the predicted probabilities support the optimal selection of variants depending on the preferences and cost functions of the researchers [2, 21].

## Conclusions

In this paper, we compared alternative variant calling pipelines, and in line with other studies [1, 3–7], we found low concordance between them, especially in the case of indels. However, we hypothesized that the intermediate annotations generated by individual variant callers are complementary information sources, which can be exploited by their automated fusion. For this task, we presented VariantMetaCaller, which utilizes the high-dimensional annotation information by fusion from multiple variant callers. As a result, it provides more accurate probabilistic scores for calls compared to earlier solutions, and thereby offers improved quantitative control for variant filtering, based on expected precision. Furthermore, the estimated probabilities can be propagated towards downstream analysis and combined with uncertainties from biological, clinical and population levels (for the incorporation of disease gene information in variant prioritization, see [22]).

The execution time of VariantMetaCaller scales quadratically and the memory footprint scales linearly with the number of variants to prioritize and is independent of the number of samples, or the coverage depth (Additional file 1: Figure S10). From typical, few hundred kilobase long gene panels up to even a few megabases, the execution time is significantly less than the run time of the variant callers. Nevertheless, Whole Genome Sequencing (WGS) data would result in an approximately 100 times increase in the number of variants compared to whole exomes. The quadratic time complexity of the SVM optimization task means that the current implementation cannot cope directly with the WGS dimensionality. Subsampling or more advanced optimization methods could be used, which is our plan for future work. However, the program is efficiently parallelized, as speedup scales linearly with the number of processing elements. Its scalability allows further computational extensions, such as the wrapping of VariantMetaCaller into an expectation-maximization framework that iteratively refines the heuristically defined variant status used for training. Another extension could be the sequential embedding of VariantMetaCaller into a Monte Carlo framework, in which the status of variants is randomly drawn according to the actually predicted probabilities. In this sense, the current work can be seen as a first step towards computationally more demanding applications. Finally, the proposed methodology is open for the dynamic adaption, replacement and incorporation of pipelines.

In summary, our study demonstrates the usefulness of intermediate information fusion, by showing that VariantMetaCaller outperforms individual variant callers and a late information fusion method under a wide range of conditions e.g. for artificially generated or real benchmark data. Our study also shows that VariantMetaCaller provides accurate probabilistic scores for calls even in areas that have been inaccessible for existing solutions, such as in targeted gene panels or organisms without accurate call sets. Thus, VariantMetaCaller broadens the application scope of quantitative, *precision-based filtering*.

## Methods

### Simulation of artificial sequencing data

We generated 100 haploid artificial human chromosomes using an in-house developed simulation software to evaluate the performance of our method. The 17th chromosome was chosen for illustrative purposes. We performed the following for each haploid chromosome: we independently selected variants (alternate alleles) based on the allele frequencies of the publicly available Exome Aggregation Consortium (ExAC) variant call set [23] (version 0.3) and modified the hg19 reference sequence to contain the chosen alternate alleles. Then we paired the haploid chromosomes to get the diploid chromosome sets of 50 artificial samples which we arranged into five distinct sample groups each containing 10 samples. The true variants of the samples served as the reference call set during the comparisons, i.e. we measured sensitivity and precision of the methods with respect to these variants.

Using the same in-house developed simulation software and the ART toolkit [24] (version: VanillaIceCream 03-11-2014) we simulated paired-end sequences (2×105 bp, mean insert size: 180, standard deviation of insert size: 10) of the exonic target regions at various read depths (coverage at exonic sites: 4,8,12,16,20,30,40,60,100 and 200). We used the Illumina sequencing platform specific error profile of ART. It is important to note that as our goal was to assess the performance of the whole pipelines (i.e. the combined effect of alignment and variant calling), the indicated coverage depths are the initial depths (before alignment) and are not necessarily equal to the depth at variant sites due to potential alignment errors or edge effects. Also note that the independently generated variants do not reflect the true linkage disequilibrium pattern of the human genome. However, as the mean distance between two neighboring variants is longer than the length of the simulated sequence reads and the variant calling algorithms currently do not utilize reference linkage information, this limitation does not affect the results.

We restricted the variant calling pipelines to the targeted regions, as the ExAC call set only covers the exonic region of the human genome.

We filtered the full length chromosome to smaller regions to demonstrate the usage of the methodology on smaller genomic regions. The resulting exonic lengths added up to approximately 100 kb, 200 kb, 300 kb and 500 kb, respectively. We selected ten non-overlapping, consecutive regions for each size.

### Real sequencing data

FASTQ files were downloaded for the sample NA12878 from a publicly available NGS data set from the Illumina base space website [25] of project: “HiSeq 2000: TruSeq PCR-Free (Platinum Genomes)”. We used only the reads from the first lane of the sequencing run.

We compared our results to the Illumina Platinum Genomes [16] (version 6.0) reference call set. This call set was assembled by Illumina Inc. in the following way: Libraries from all 17 samples in the CEPH pedigree trio 1463 (including the sample NA12878) were prepared using the TruSeq DNA PCR-Free Library Prep Kit and sequenced at 50 × coverage depth on a HiSeq 2000 System. Several different analysis pipelines were used to call variants for all pedigree members (including Isaac, BWA+GATK, BWA+FreeBayes, Cortex and CGI pipelines). Variant calls were analyzed with a specialized workflow that accounts for the inheritance structure and concordance across the different methods. We used the resulting publicly available high-confidence variant calls (VCF) and confident regions (BED files) as “gold standard” during the evaluations. Further details of the analysis workflow can be obtained on the Illumina Platinum Genomes website [16].

### Variant calling pipelines

We aligned the quality filtered (with PRINSEQ [26] version 0.20.4) sequencing reads to the hg19 reference genome with BWA–MEM [27] using default parameters or, alternatively, with Bowtie 2 [28] using *very-sensitive* default settings. We applied GATK [29] base quality score recalibration and indel realignment only to the real sequencing data.

We used four variant callers to detect SNPs and short indels: GATK UnifiedGenotyper and HaplotypeCaller [29] (version 3.3-0), FreeBayes [30] (version v0.9.20-17-g5f1bc44-dirty) and SAMtools combined with BCFtools [31] (version 1.1-22-gc61d8d1). We used all variant callers with default parameters with the following exception: FreeBayes was set to ignore multi-nucleotide polymorphisms and complex events in order to ease the combination of the variants calls.

Furthermore, variant call sets were left aligned using BCFtools in order to unify different representations of the same variants.

Hard filters were set according to the GATK Best Practices recommendations [32] for GATK called variant sets. For FreeBayes and SAMtools we set the quality threshold to 30 and 100 as a hard filter for real and artificial sequencing data, respectively.

Note that recent extensions of variant calling pipelines, such as read correction [33–35] are not covered in our study.

### Support Vector Machine-based combination of variant callers

#### Support Vector Machine

VariantMetaCaller combines unfiltered, multi- or single-sample variant calls of individual variant callers by the following way:

First, we merge variants called by the individual variant callers and unify overlapping variant calls with different allele representations. Next, we train an SVM for SNPs and indels and for each input variant caller separately.

We use the following heuristics to define the positive training instances: we initialize a threshold *t* to be the number of the variant callers (or a number defined by the user at program start). Then, the positive training set consists of variants simultaneously called by at least *t* callers, with an additional constraint that all callers must contribute. If any of these conditions fail, the threshold is decreased until a proper positive set is established.

Then, for each variant caller we determine the singly-called variants (i.e. variants found at most by one or a user-defined number of callers). If all callers have singly-called variants then these serve as negative training instances and a two-class SVM is used. If there is at least one caller that has no singly-called variant then for each variant caller a one-class SVM is trained using only the positive training instances (e.g. this is dominant in the case of the restricted smaller genomic regions). Finally, we train an SVM classifier for each data set.

Note that the present application of SVM deviates from the standard frequentist, machine-learning applications of SVMs which typically assume independent, identically distributed (i.i.d.) data, perfect class labels and a cross-validation framework for the evaluation of future performance. However, in genomic applications, variants are not i.i.d., class labels are heuristics, reference class labels are used to estimate the performance on the actual data, and multiple reference data sets are used for statistical evaluation in case of simulated data sets.

We use a modified version of LIBSVM [36] to implement SVM functionality.

##### Computing probability of variants

Given a variant caller, we compute the conditional probability of each variant being a “real” variant (i.e. belonging to the positive class) [37], where callers have equal probabilities. Then, the final score is the probability of variants marginalized over all variant callers. Thus, for the *i*th variant:

(1) $$ P_{SVM}(i) = \frac{1}{N}\sum\limits_{j=1}^{N}Pr(y_{ij}=1|x_{ij}),  $$

where N equals the number of variant callers, *Pr*(*y*_*ij*_=1|*x*_*ij*_) is the probability of the *i*th variant being in the positive class in case of the *j*th variant caller.

Additionally, we order the variants by *P*_*SVM*_ decreasingly, and compute the estimated precision for each *i* index along the ordering by the following formula:

(2) $$ \begin{aligned} E_{PREC}^{(i)} &= \frac{TP^{(i)}}{TP^{(i)}+FP^{(i)}} \\ &= \frac{\sum_{j=1}^{i}P_{SVM}^{(j)}}{\sum_{j=1}^{i}P_{SVM}^{(j)} + \sum_{j=1}^{i}(1-P_{SVM}^{(j)})} \\ &= \frac{1}{i}\sum\limits_{j=1}^{i}P_{SVM}^{(j)}, \end{aligned}  $$

where *TP*^(*i*)^ and *FP*^(*i*)^ are the estimated number of true positives and false positives, respectively, along the ordering of the variants at the *i*th index. Note, that the estimated false discovery rate (FDR) is $E_{\textit {FDR}}^{(i)} = 1-E_{\textit {PREC}}^{(i)}$.

##### Adjusting the estimated probabilities

We found that in case of the simulated chromosome, the expected precisions were underestimated, which requires further investigation (for the probabilistic interpretation of SVM’s output, see [38, 39]). However, the rate of the real versus estimated precisions could be properly captured by the coverage depth and the choice of the aligner software (Additional file 1: Figure S11). We fitted exponential functions to the empirical observations, and in case of the real data set we used the value of these functions to adjust the estimated probabilities in order to adjust the estimated precision.

##### Effects of noise in training data

To investigate the presence of noise in the constructed positive and negative training instances, we calculated the fraction of true variants in the negative samples and the fraction of false variants in the positive samples (Additional file 1: Figure S1). Because of the relatively high error rates in the negative samples, we calculated the performance gain of the “ideally trained” model (i.e. using only the true negatives and true positives for training), which showed a significant, but modest increase in performance (mean AUPRC gain: <0.007 *%* for SNPs and <0.4 *%* for indels, Additional file 1: Figure S2).

#### Parameters for Support Vector Machine

We use the radial basis function (RBF) kernel for SVM. A two-level grid search together with 5-fold cross validation is applied to determine the penalty *C* and the *γ* parameter for each RBF kernel leading to the highest accuracy. The first level of the grid search iterates through 2^−5^ to 2^17^ by 2^2^ for the parameter *C*, and through 2^−17^ to 2^3^ by 2^2^ for the parameter *γ*. At the second level, a finer grid search (by 2^0.2^) is performed on the region that gave the highest accuracy during cross-validation. After the best (*C*,*γ*) is found, the whole training set is used again to compute the final model.

The grid search is parallelized with OpenMP. The parameters of the grid search are configurable.

#### Input features

The program creates a data set for each variant caller from the available annotations generated by the caller. Besides using standard annotations (e.g. read depth, variant quality, mapping quality etc.) the program calculates additional features such as the number of bases to the closest variant, mean and standard deviation of the entropy of the genotype distribution across all samples of the variant, entropy of the reference sequence near the variant. The full listing of the utilized annotations and their short description can be found in Additional file 2. The features and their transformation and scaling methods can be fully configured using a configuration file of the program.

#### Combined genotypes

The final genotype probabilities are calculated based on the genotype probabilities computed by the variant callers. Genotypes with maximal probabilities are indicated in the resulting variant call format (VCF) file.

### Comparisons of the methods

We compared the performance of the variant callers and VariantMetaCaller by plotting precision-recall curves. First, for each variant caller, we ordered the hard filtered variants first by decreasing quality, and in case of ties in the qualities: by decreasing read depth. For VariantMetaCaller, we ordered the combined variants first by decreasing SVM score and then by decreasing mean read depth. Next, we computed the precision and the sensitivity (i.e. recall) at each threshold of variant quality and SVM score, respectively, and plotted precision by sensitivity.

We calculated the area under the precision-recall curve (AUPRC) as well, using trapezoid rule integration [40], and we calculated the differences between the AUPRC values of the variant callers and VariantMetaCaller.

### Software description and requirements

The VariantMetaCaller software is written in C++. It is compiled into a command line tool, and can work on Linux/Unix systems. The software expects standard VCF files as input and produces a VCF file as output which includes all variants from input files and SVM scores for each variant as additional annotations. The available parametrization is described in a help screen and on the software’s website. The computations described in the paper were performed on a standard desktop PC. For a detailed analysis of computational requirements see Conclusions and Additional file 1: Figure S10.

## Availability of supporting data and software

The VariantMetaCaller software and the artificial sequencing data sets supporting the results of this article is freely available at http://bioinformatics.mit.bme.hu/VariantMetaCaller/. The real sequencing data set is available (after registration) as part of a public Illumina BaseSpace project: “HiSeq 2000: TruSeq PCR-Free (Platinum Genomes)” at https://basespace.illumina.com/home/index.

## Additional files

Additional file 1
**Supplementary results, figures and tables.** Supplementary results, **Figures S1–S11** and **Tables S1–S6**.

Additional file 2
**Variant annotations used as features for SVMs.** The full listing and short description of the variant annotations used as features for Support Vector Machines.

## Abbreviations

AUPRC: Area under the precision–
recall curve; AUROC: Area under the receiver operating characteristic curve
CI: Confidence interval
ExAC: Exome Aggregation Consortium
FDR: False discovery rate
GATK: Genome analysis Toolkit
i.i.d.: Independent, identically distributed
MAE: Mean absolute error
NGS: Next–
generation sequencing; RBF: Radial basis function
SNP: Single nucleotide polymorphism
SVM: Support Vector Machine
VCF: Variant call format
VQSR: Variant quality score recalibration
