# Supplementary material for: VariantMetaCaller: automated fusion of variant calling pipelines for quantitative, precision-based filtering
Source: BMC Genomics. 2015 Oct 28;16:875. doi: 10.1186/s12864-015-2050-y (PMC4625715; doi:10.1186/s12864-015-2050-y)
Supplement: Additional file 1 — Supplementary results, figures and tables. Supplementary results, Figures S1–S11 and Tables S1–S6. [file 12864_2015_2050_MOESM1_ESM.pdf]

SUPPLEMENTARY RESULTS, FIGURES AND TABLES OF: VARIANTMETACALLER: AUTOMATED FUSION OF VARIANT CALLING PIPELINES FOR QUANTITATIVE, PRECISION-BASED FILTERING

ANDRÁS GÉZSI[1], BENCE BOLGÁR[2], PÉTER MARX[2], PETER SARKOZY[2], CSABA SZALAI[1] AND PÉTER ANTAL[2]

[1]*Department of Genetics, Cell- and Immunobiology, Semmelweis University*

[2]*Department of Measurement and Information Systems, Budapest University of Technology and Economics*

E-mail address: gezsi.andras@gmail.com.

Supplementary results and discussion

Performance of individual variant callers on simulated sequencing data. We systematically assessed the performance of the individual variant callers by computing the sensitivity and precision of the unfiltered call sets, particularly assessing the impact of coverage depth.

The sensitivity of the variant callers increased with increasing coverage depth in most cases, reflecting the general expectation that more information at variant sites is beneficial for variant calling [1]. However, in case of indels, the sensitivity of SAMtools decreased with increasing coverage at high depths ($> 60\times$ coverage), independently of the aligner, which needs further investigation.

In case of SNPs, SAMtools provided the highest sensitivity at low to medium coverage depths regardless of the aligner, and for coverages above $100\times$, UnifiedGenotyper in case of BWA alignment and FreeBayes in case of Bowtie 2 alignment produced the highest sensitivity (Table S2, Figure S5A). The variant caller that provided the second highest sensitivity varied with coverage depth, but in most cases, especially at low depths, FreeBayes performed better than the GATK based variant callers. In case of indels, the sensitivity of HaplotypeCaller was the highest at all depths, independently of the aligner (Table S2, Figure S5B). At low coverage depths, FreeBayes, and above $16\times$ coverage, UnifiedGenotyper provided the second highest sensitivity.

All variant callers use an inner cutoff for reporting variant sites, and these thresholds vary between methods. As the relative superiority in terms of sensitivity may result in losing precision, it is also important to investigate the precision of the variant calls in case of each method. The calculated precisions showed that HaplotypeCaller provided the most precise SNP calls, and UnifiedGenotyper provided the most precise indel calls in our experiments, regardless of the aligner. In order to better visualize the precision, we calculated its complement, the false discovery rate, i.e. the fraction of false variants called by each method (Figure S6). In case of SNPs, SAMtools called less and UnifiedGenotyper called more erroneous variants with increasing coverage, while the error rates of HaplotypeCaller and FreeBayes were relatively stable compared to the other two methods. In case of indels, the rate of false variants increased strongly with increasing coverage for SAMtools compared to the other methods, and the fraction of false variants was approximately $2 - 4\times$ greater for SAMtools than for the other variant callers. Our results partly agree with the work of Liu *et al.* who found that the precision of SAMtools outperformed that of UnifiedGenotyper and the sensitivity of UnifiedGenotyper outperformed that of SAMtools at all depths [2]. In our case, the former was true only for SNPs and only at high read depths, and the latter was true only at high depths. However, they used older versions of the variant callers.

The increasing false discovery rate with increasing coverage is probably due to the accumulation of noise at each position, and typically methods that call variants on a per-locus basis, like SAMtools and UnifiedGenotyper, seem to be more prone to this type of error.

In summary, there was no best method with superior sensitivity and precision at all read depths, even concerning SNPs or indels, although HaplotypeCaller performed quite well in case of indels and was the most precise in case of SNPs.

The sensitivity and the precision of the variant callers were markedly higher for SNPs than for indels at the same coverage depths (Table S2). This is in line with other studies which also found that current indel calling methods are relatively inaccurate and imprecise [3], in spite of the fact that indels potentially have a greater effect on gene products than SNPs [4]. The difficulty of indel calling may arise from multiple sources: (1) sequence reads covering indels are generally more difficult to map since a gapped alignment is needed [5, 6], (2) the position of an indel with respect to the reference sequence is usually ambiguous and in theory, indels can be represented by at any of multiple locations [3]. The first problem may be solved by increasing coverage depth or read length (for indels with large insert sizes) [7] but our results show that a significant increase is needed to achieve the same sensitivity levels as of SNPs (for example 200× coverage for indels and 16× coverage for SNPs provides approximately equal sensitivity in case of HaplotypeCaller). The problem of ambiguous representations can be party solved by left-justifying the indel within its possible locations (also known as left normalization), but this does not cover all problematic scenarios.

We note that in this analysis the reference variants are based on the ExAC call set which was called by HaplotypeCaller. Therefore, there may be a bias in a sense that variants specific to HaplotypeCaller may be overrepresented, and variants specific to the other variant callers may be underrepresented in the reference call set, particularly in case of indels. Therefore, the relative superiority of HaplotypeCaller should be treated with caution and needs further investigation using e.g. randomly generated indels.

Effects of the aligner. The variant callers generally achieved higher maximum sensitivity when BWA, as opposed to Bowtie 2, was used and the differences were statistically significant (Table S4). The highest difference was observed in case of HaplotypeCaller (mean difference: 0.057 and 0.053 for SNPs and indels, respectively), although the precision was less using BWA, as opposed to Bowtie 2, alignments. In case of the other variant callers the mean sensitivity difference was somewhat smaller (range: $0.031 - 0.042$), but altogether the precision difference was also positive. These results are in compliance with former findings [8, 9]. Highnam *et al.* reported higher precision rate and sensitivity using BWA compared to Bowtie 2 using the same variant caller method, UnifiedGenotyper [8].

Impact of hard filters on individual variant callers. Hard filters are extensively used for improving precision of variant calls [10, 11, 12]. However, as there is no direct score or combination of scores that clearly separates true variants from erroneously called variants, the precision and sensitivity are inversely related to each other, and one can improve precision only at the price of losing some sensitivity. We applied hard filters to the individual variant callers based on the current recommendations (see Methods), bearing in mind, that these are not directly optimal and would need experimenting. We also note that the precision of the unfiltered call sets was generally high, especially in the case of SNPs, and therefore the expected precision gain by applying hard filters was low.

The effect of hard filters was considerably different for the individual variant callers (Figure S7). In case of SNPs, the effects of hard filtering on the results of HaplotypeCaller was relatively low and independent of coverage depth. For UnifiedGenotyper, a

significant sensitivity loss was observed for increasing coverage depths while the precision increased only a little. Hard filtering for SAMtools and FreeBayes showed similar characteristics, considerably decreasing sensitivity in favour of very small increase in precision and the sensitivity loss was larger for high coverages. The only exception was observed at very low coverage depth for SAMtools where the precision gain was relatively higher (0.03), at the expense of high sensitivity loss (0.3). In case of indels, hard filtering strategy performed moderately well for SAMtools and HaplotypeCaller, although the sensitivity loss generally surpassed the precision gain in case of SAMtools. For UnifiedGenotyper, hard filtering had very low effect, and for FreeBayes, the effect was contradictory, as the precision also decreased.

The coverage dependency of hard filtering observed in case of SAMtools and FreeBayes can be explained by the simple filtering rule, as we used only the quality estimation of the variants with a predefined threshold for filtering (see Methods). Evidently, the quality scores of the variants increase with increasing depth, and we filter less and less variants.

In summary, our results show that the utility of hard filtering is limited in our case and that the same hard filter settings are not appropriate for all coverage depths.

References

[1] Pirooznia, M., Kramer, M., Parla, J., Goes, F.S., Potash, J.B., McCombie, W.R., Zandi, P.P.: Validation and assessment of variant calling pipelines for next-generation sequencing. Human genomics **8**(1), 14 (2014). doi:10.1186/1479-7364-8-14

[2] Liu, X., Han, S., Wang, Z., Gelernter, J., Yang, B.-Z.: Variant callers for next-generation sequencing data: a comparison study. PloS one **8**(9), 75619 (2013). doi:10.1371/journal.pone.0075619

[3] O'Rawe, J., Jiang, T., Sun, G., Wu, Y., Wang, W., Hu, J., Bodily, P., Tian, L., Hakonarson, H., Johnson, W.E., Wei, Z., Wang, K., Lyon, G.J.: Low concordance of multiple variant-calling pipelines: practical implications for exome and genome sequencing. Genome medicine **5**(3), 28 (2013). doi:10.1186/gm432

[4] Zhao, H., Yang, Y., Lin, H., Zhang, X., Mort, M., Cooper, D.N., Liu, Y., Zhou, Y.: DDIG-in: discriminating between disease-associated and neutral non-frameshifting micro-indels. Genome biology **14**(3), 23 (2013). doi:10.1186/gb-2013-14-3-r23

[5] Li, H., Durbin, R.: Fast and accurate short read alignment with Burrows-Wheeler transform. Bioinformatics (Oxford, England) **25**(14), 1754–60 (2009). doi:10.1093/bioinformatics/btp324

[6] Krawitz, P., Rödelsperger, C., Jäger, M., Jostins, L., Bauer, S., Robinson, P.N.: Microindel detection in short-read sequence data. Bioinformatics (Oxford, England) **26**(6), 722–9 (2010). doi:10.1093/bioinformatics/btq027

[7] Neuman, J.A., Isakov, O., Shomron, N.: Analysis of insertion-deletion from deep-sequencing data: software evaluation for optimal detection. Briefings in bioinformatics **14**(1), 46–55 (2013). doi:10.1093/bib/bbs013

[8] Highnam, G., Wang, J.J., Kusler, D., Zook, J., Vijayan, V., Leibovich, N., Mittelman, D.: An analytical framework for optimizing variant discovery from personal genomes. Nature communications **6** (2015)

[9] Hatem, A., Bozdağ, D., Toland, A.E., Çatalyürek, Ü.V.: Benchmarking short sequence mapping tools. BMC bioinformatics **14**(1), 184 (2013)

[10] Yu, X., Sun, S.: Comparing a few SNP calling algorithms using low-coverage sequencing data. BMC bioinformatics **14**(1), 274 (2013). doi:10.1186/1471-2105-14-274

[11] Li, H.: Toward better understanding of artifacts in variant calling from high-coverage samples. Bioinformatics (Oxford, England), 356 (2014). doi:10.1093/bioinformatics/btu356

[12] Park, M.-H., Rhee, H., Park, J.H., Woo, H.-M., Choi, B.-O., Kim, B.-Y., Chung, K.W., Cho, Y.-B., Kim, H.J., Jung, J.-W., Koo, S.K.: Comprehensive analysis to improve the validation rate

for single nucleotide variants detected by next-generation sequencing. PloS one **9**(1), 86664 (2014). doi:10.1371/journal.pone.0086664

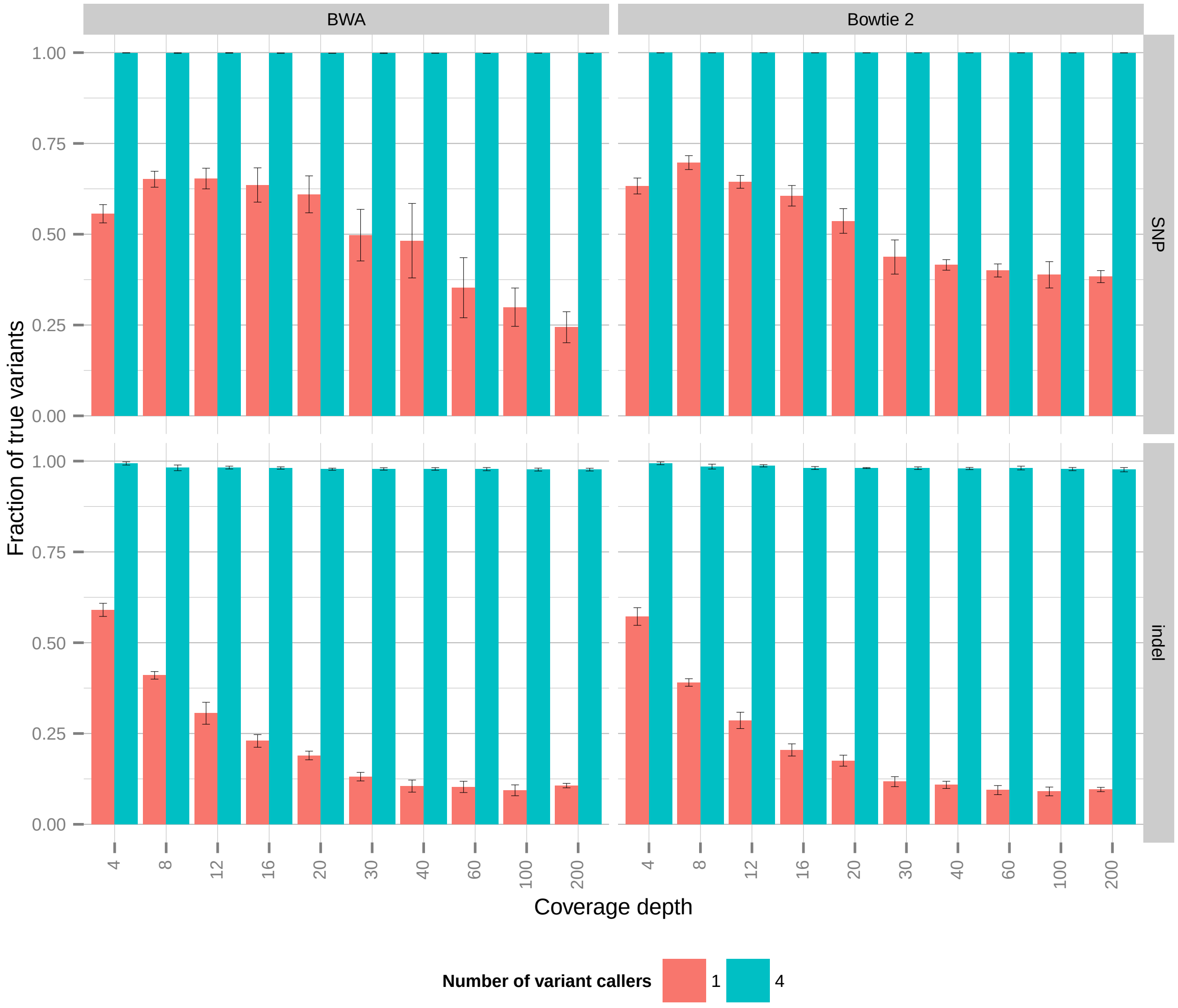

Figure S1. **Fraction of true variants among variants called by one or all variant callers in case of the simulated chromosome.** Sequencing reads covering the exonic region of a selected chromosome were simulated for 50 artificially generated samples with pre-known variations to the human genome (i.e. reference variants). Variants were called on the BWA–MEM and Bowtie 2 aligned reads by HaplotypeCaller, UnifiedGenotyper, FreeBayes and SAMtools on five sample groups each containing ten samples. Bars represent the fraction of true variants among variants called by one (red) or all four (blue) variant callers at various coverage depths. Error bars represent 95% confidence intervals. Top row: SNPs, bottom row: indels, left column: BWA alignment, right column: Bowtie 2 alignment.

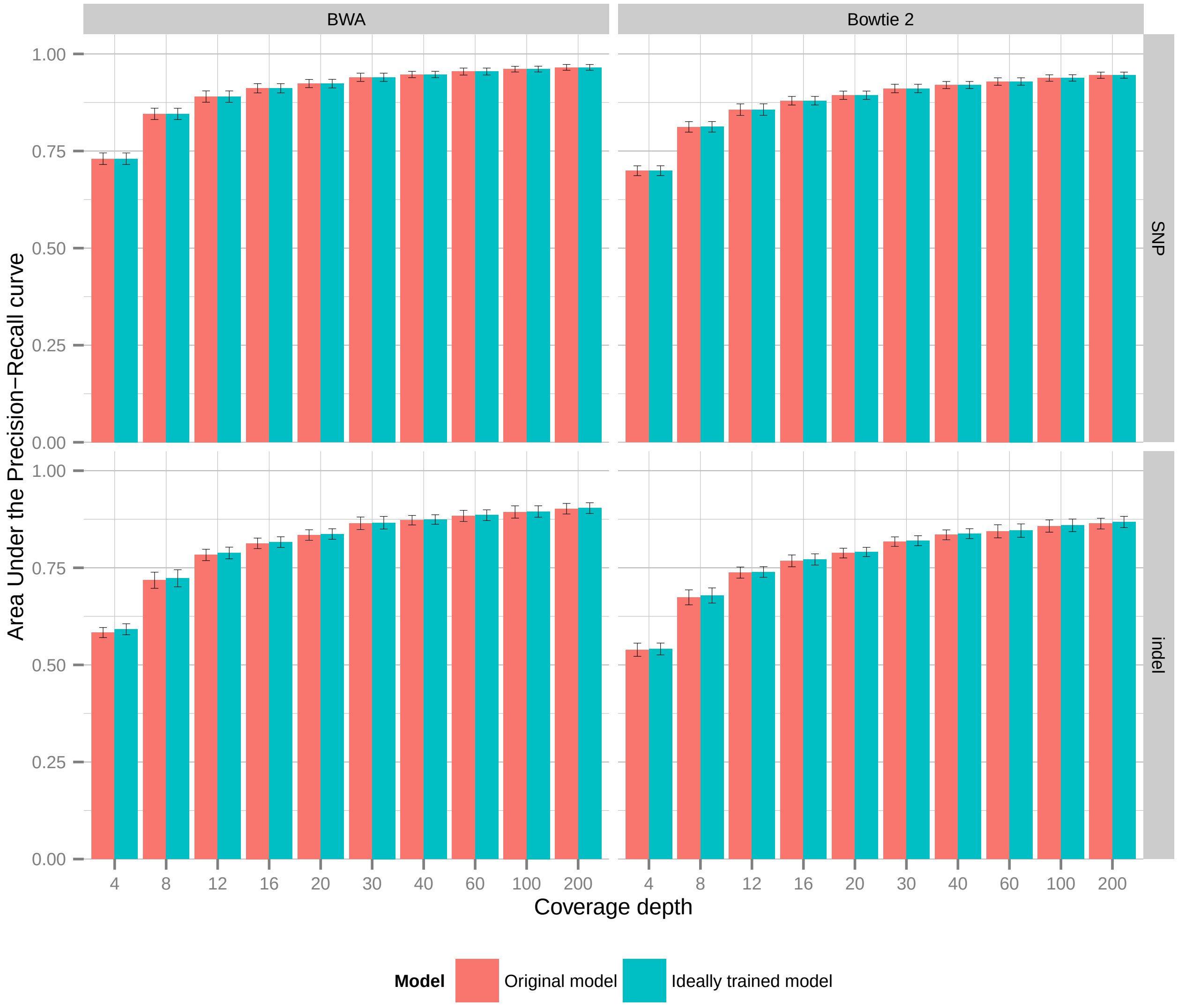

Figure S2. **Area under the precision-recall curves of the original and ideally trained SVMs in case of the simulated chromosome.** As the training instances used for training the core SVMs of VariantMetaCaller contains noise (see Figure S1), we calculated the area under the precision-recall curves for "ideally trained" models (blue) and compared them to the original models (red). In case of the ideally trained models, only the true negative and true positive variants were used for training the SVMs. Error bars represent 95% confidence intervals. Top row: SNPs, bottom row: indels, left column: BWA alignment, right column: Bowtie 2 alignment. See caption of Figure S1 or Methods for the description of the simulated data set.

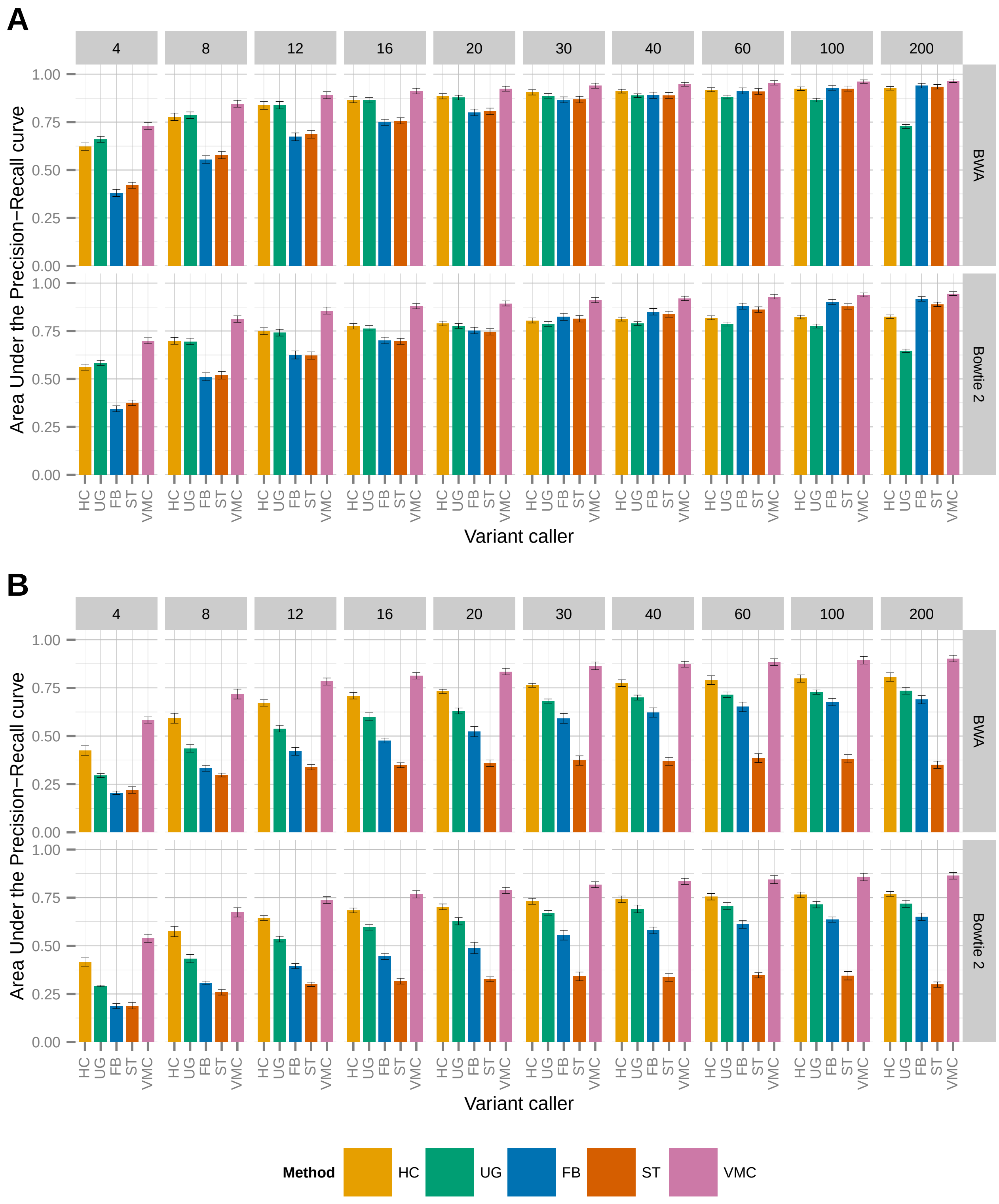

Figure S3. **Area under the precision-recall curves in case of the simulated chromosome.** The area under the precision-recall curves was calculated for hard filtered call sets of four individual variant callers and for the results of VariantMetaCaller for SNPs (**A**) and for indels (**B**). The rows differentiate between alignment algorithms and the columns represent different coverage depths. Variant calling was performed on five sample groups each containing ten samples. Error bars represent 95% confidence intervals of AUPRC based on the results of the different sample groups. See caption of Figure S1 or Methods for the description of the simulated data set. FB = FreeBayes, HC = HaplotypeCaller, ST = SAMtools, UG = UnifiedGenotyper, VMC = VariantMetaCaller

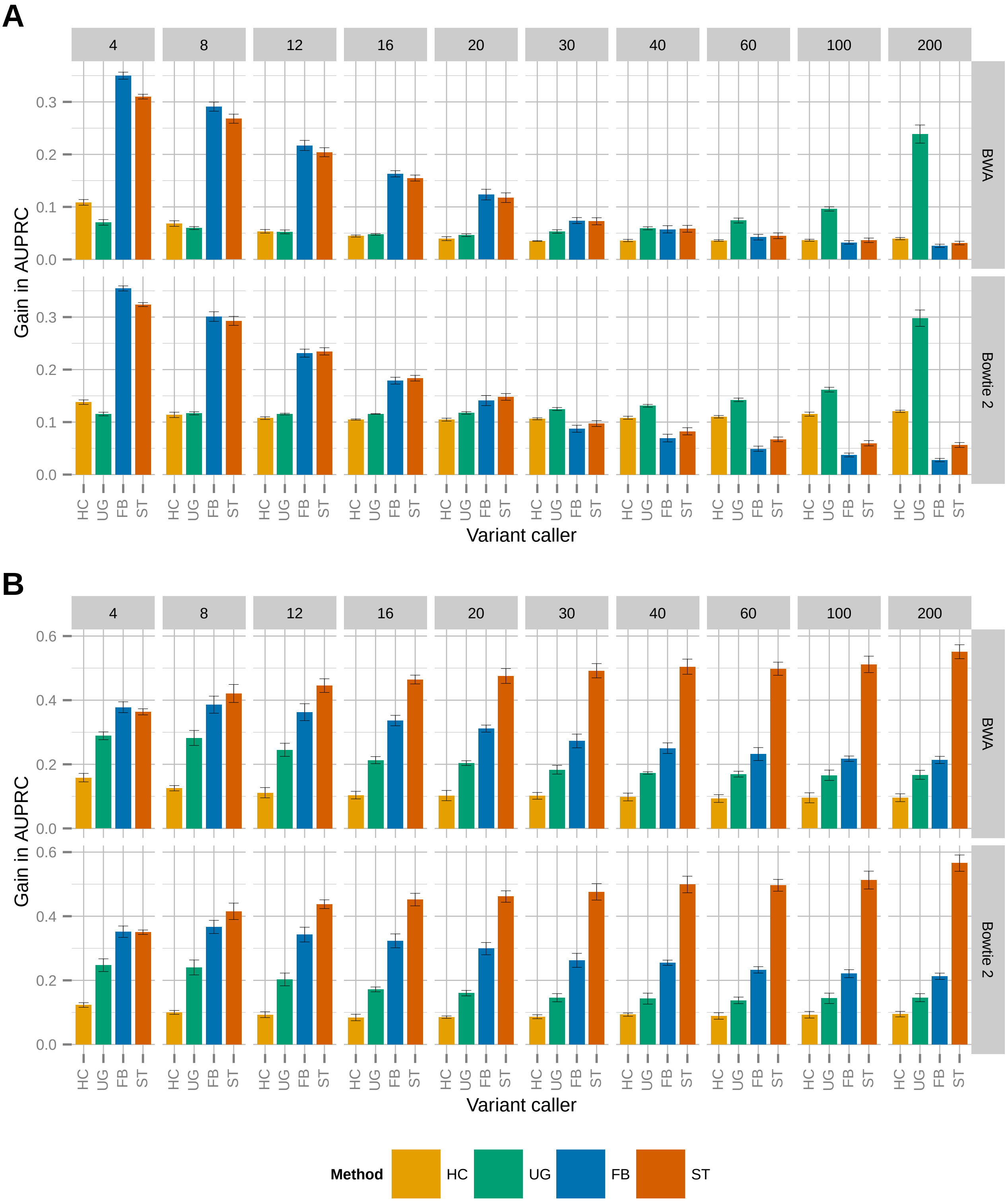

FIGURE S4. **Differences of area under the precision-recall curves between VariantMetaCaller and each variant caller in case of the simulated chromosome.** The difference between the area under the precision-recall curves for VariantMetaCaller and each variant caller was calculated for SNPs (**A**) and for indels (**B**). The rows differentiate between alignment algorithms and the columns represent different coverage depths. Variant calling was performed on five sample groups each containing ten samples. Error bars represent 95% confidence intervals of the difference of AUPRC measures based on the results of the different sample groups. See caption of Figure S1 or Methods for the description of the simulated data set. FB = FreeBayes, HC = HaplotypeCaller, ST = SAMtools, UG = UnifiedGenotyper

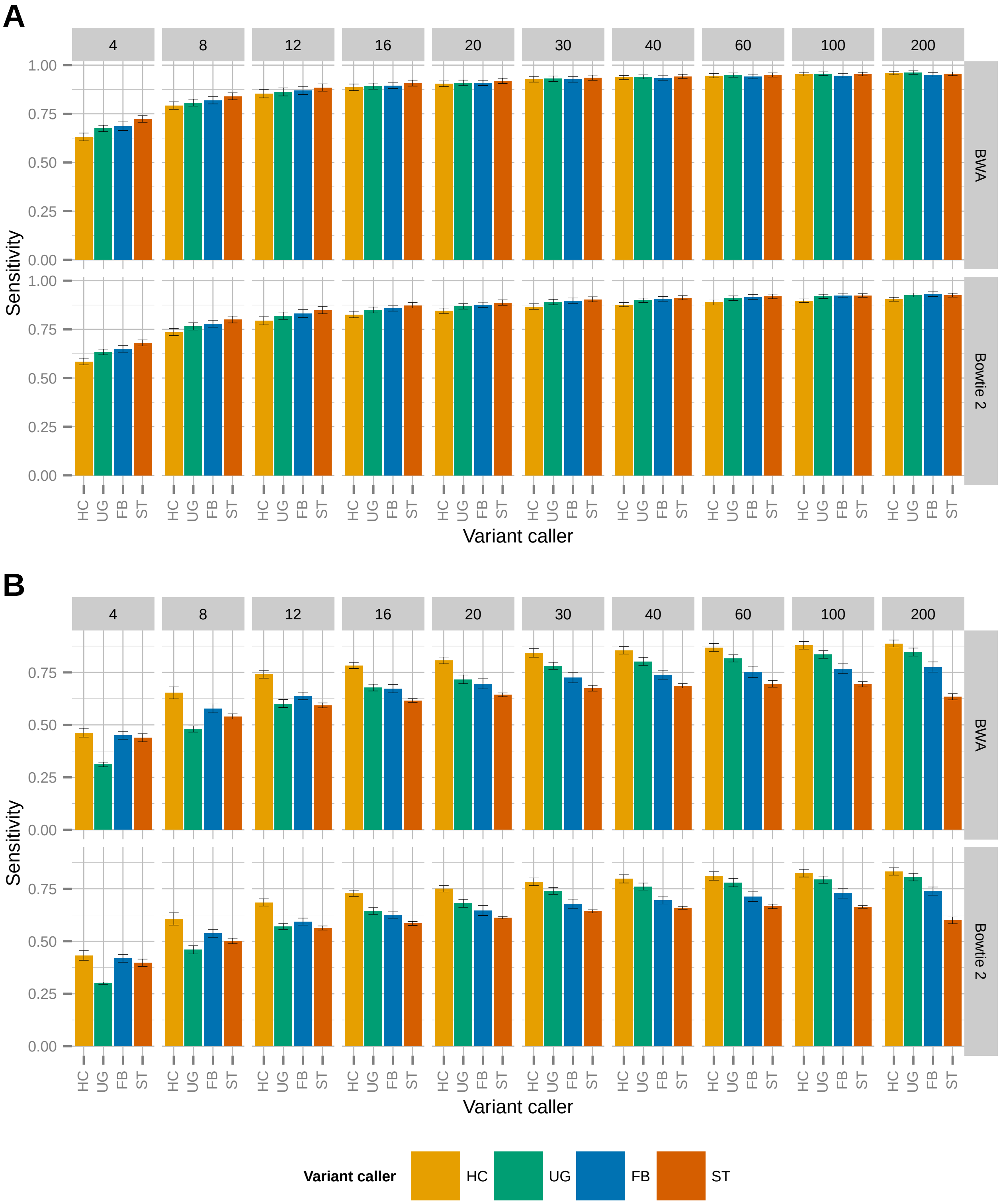

Figure S5. **Sensitivity of the individual variant callers in case of the simulated chromosome.** Bars represent the sensitivity of the unfiltered SNPs (**A**) and indels (**B**) by each method with respect to the reference variants. The rows differentiate between aligners and the columns represent different coverage depths. Error bars represent 95% confidence intervals of the estimate. See caption of Figure S1 or Methods for the description of the simulated data set. FB = FreeBayes, HC = HaplotypeCaller, ST = SAMtools, UG = UnifiedGenotyper

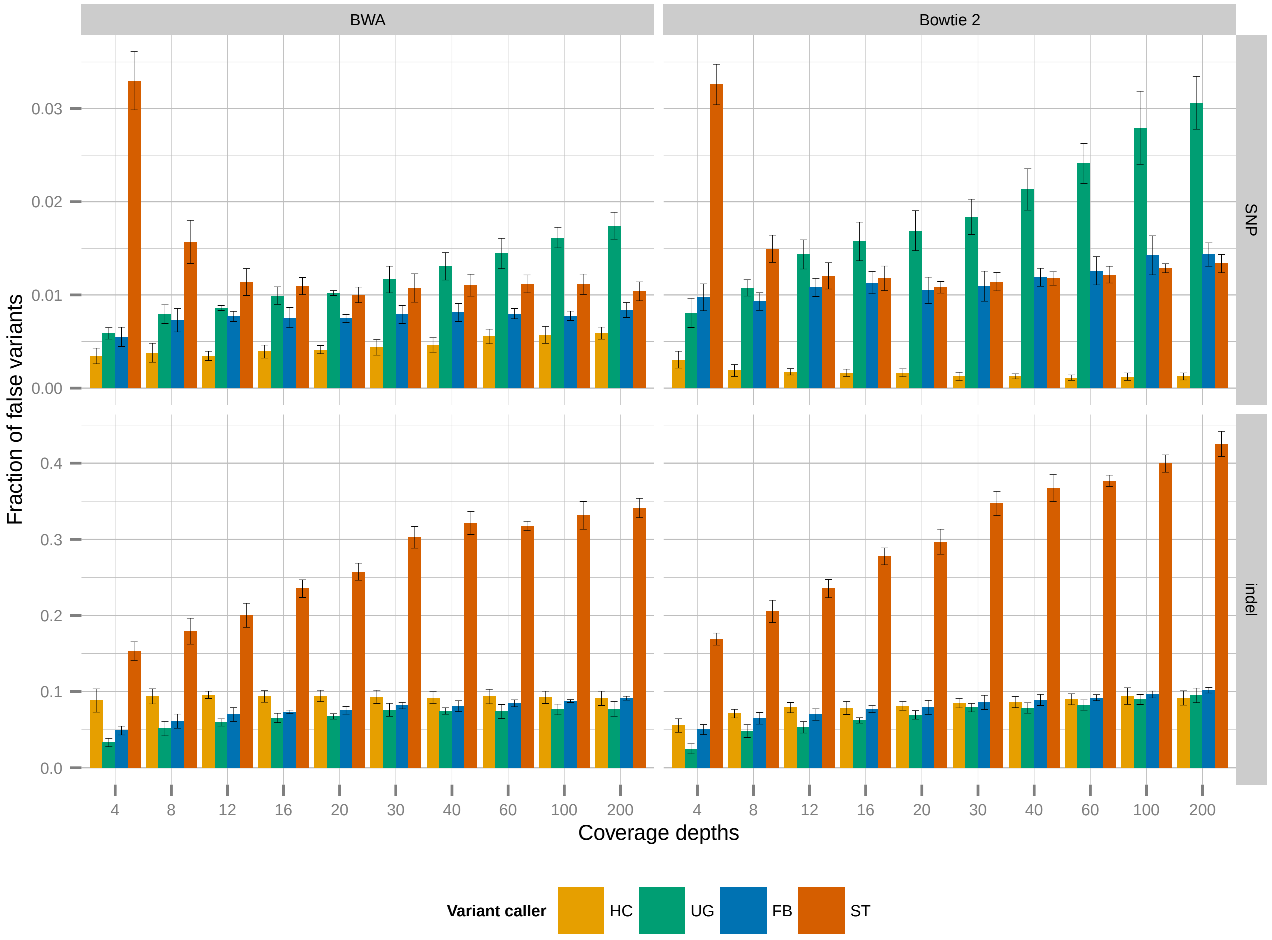

FIGURE S6. **Fraction of falsely called variants by each individual variant caller in case of the simulated chromosome.** Bars represent the fraction of erroneously called SNPs (top row) and indels (bottom row) by each individual variant caller with respect to the reference variants based on BWA (left) and Bowtie 2 (right) alignments, respectively. Error bars represent 95% confidence intervals. See caption of Figure S1 or Methods for the description of the simulated data set. FB = FreeBayes, HC = HaplotypeCaller, ST = SAMtools, UG = UnifiedGenotyper

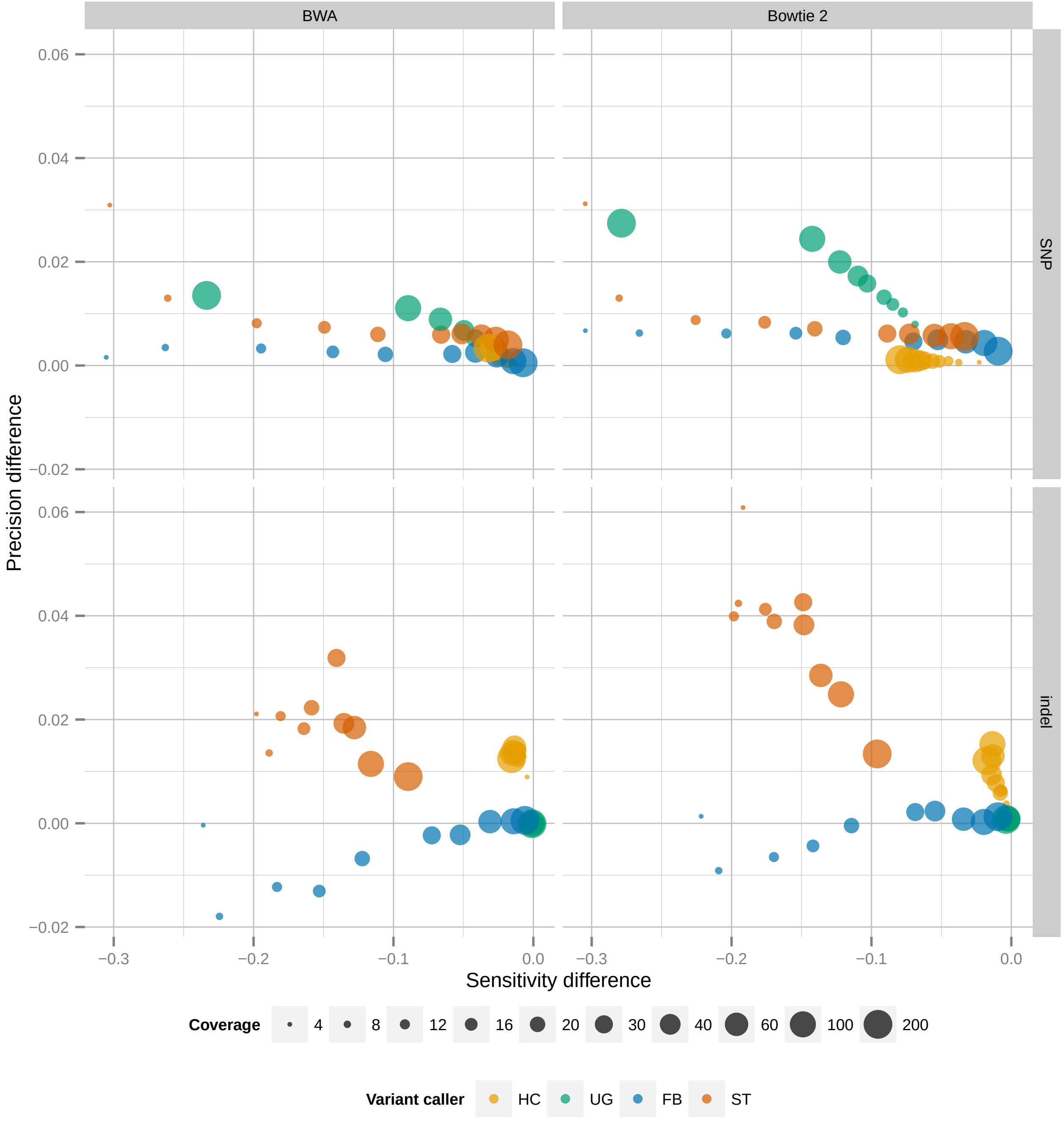

FIGURE S7. **Effect of hard filters to the sensitivity and precision of individual variant callers in case of the simulated chromosome.** Points represent the mean change (averaged over different sample groups) in sensitivity and precision by applying hard filters. The size of the point is proportional to the coverage depth. Top row: SNPs, bottom row: indels, left column: BWA alignment, right column: Bowtie 2 alignment. See caption of Figure S1 or Methods for the description of the simulated data set. FB = FreeBayes, HC = HaplotypeCaller, ST = SAMtools, UG = UnifiedGenotyper

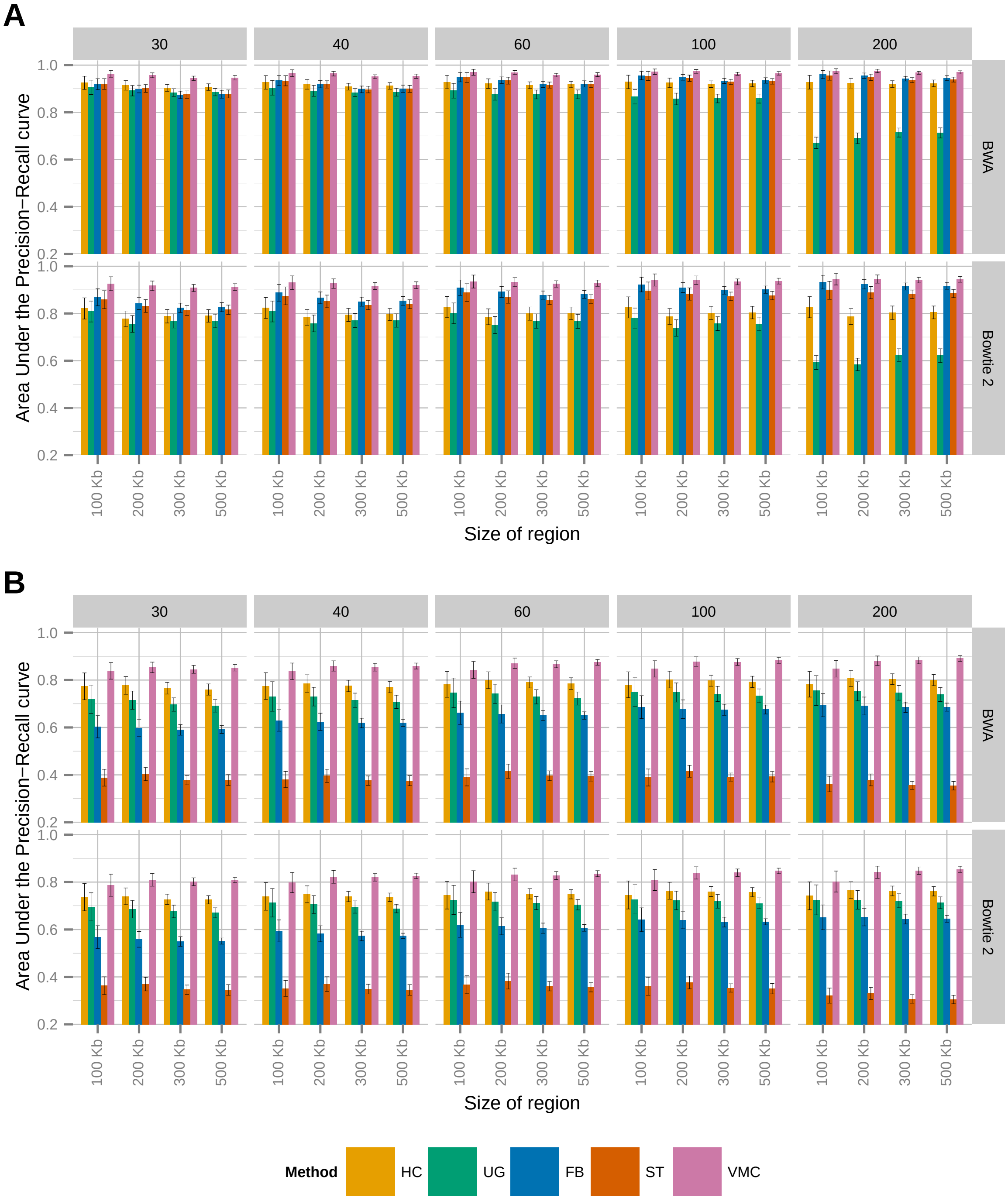

Figure S8. **Area under the precision-recall curves in case of the reduced target regions of the simulated chromosome.** See caption of Figure S1 or Methods for the description of the simulated data set. The full length simulated chromosomes were filtered to smaller non-overlapping regions, where the exonic length added up to approximately 100 kb, 200 kb, 300 kb and 500 kb, respectively. Ten regions were selected for each size and the analyses were performed on each region. The area under the precision-recall curves was calculated for hard filtered call sets of the four individual variant callers and for the results of VariantMetaCaller for SNPs (**A**) and for indels (**B**). The rows differentiate between alignment algorithms and the columns represent different coverage depths. Variant calling was performed on five sample groups each containing ten samples. Error bars represent 95% confidence intervals of AUPRC based on the results of the different sample groups and the different regions of the same size. FB = FreeBayes, HC = HaplotypeCaller, ST = SAMtools, UG = UnifiedGenotyper, VMC = VariantMetaCaller

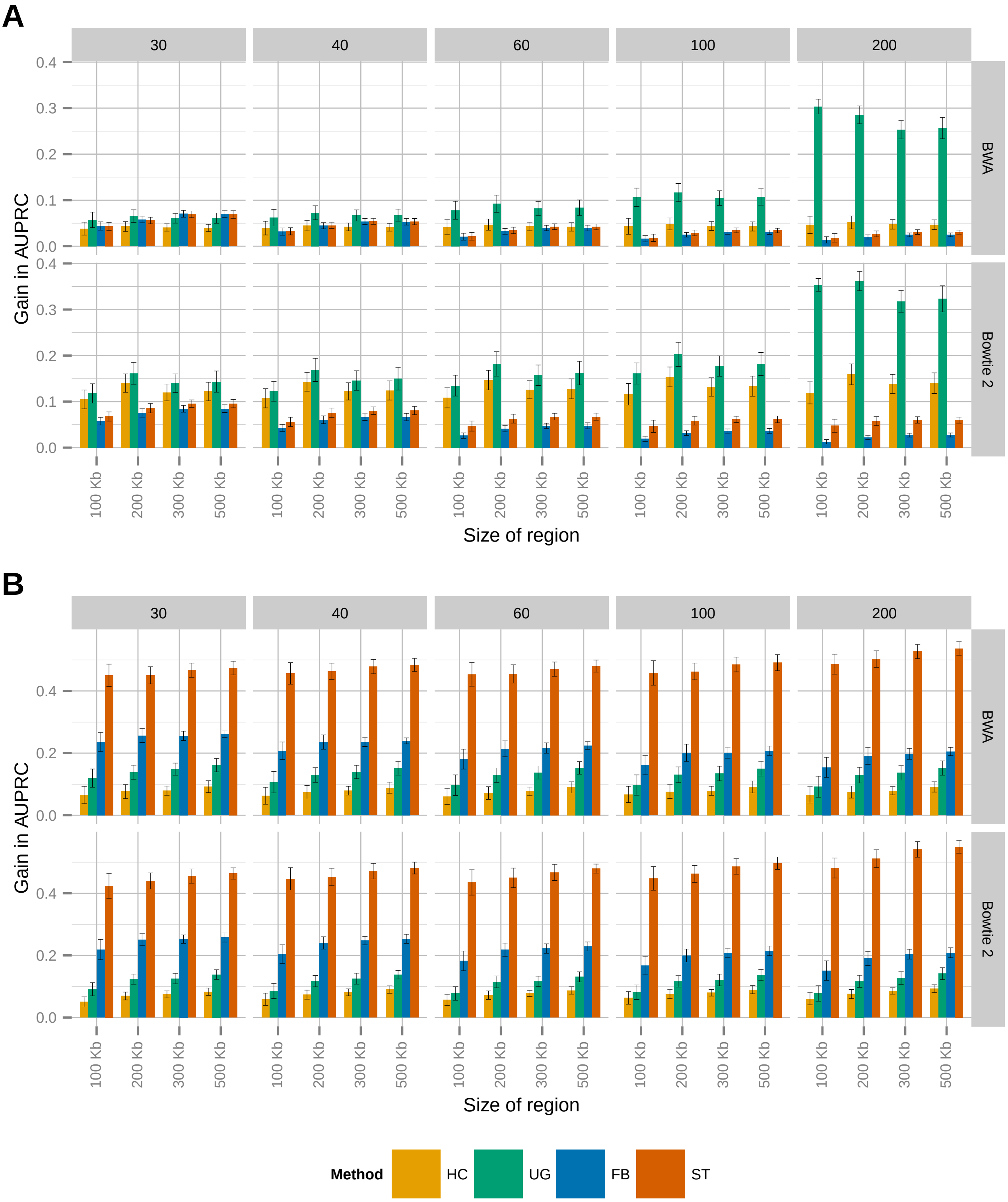

FIGURE S9. **Differences of area under the precision-recall curves between VariantMetaCaller and each variant caller in case of the reduced target regions of the simulated chromosome.** See caption of Figure S1 and S6 or Methods for the description of the simulated data set. The difference between the area under the precision-recall curves for VariantMetaCaller and each variant caller was calculated for SNPs (**A**) and for indels (**B**). The rows differentiate between alignment algorithms and the columns represent different coverage depths. Variant calling was performed on five sample groups each containing ten samples. Error bars represent 95% confidence intervals of the difference of AUPRC measures based on the results of the different sample groups and the different regions of the same size. FB = FreeBayes, HC = HaplotypeCaller, ST = SAMtools, UG = UnifiedGenotyper

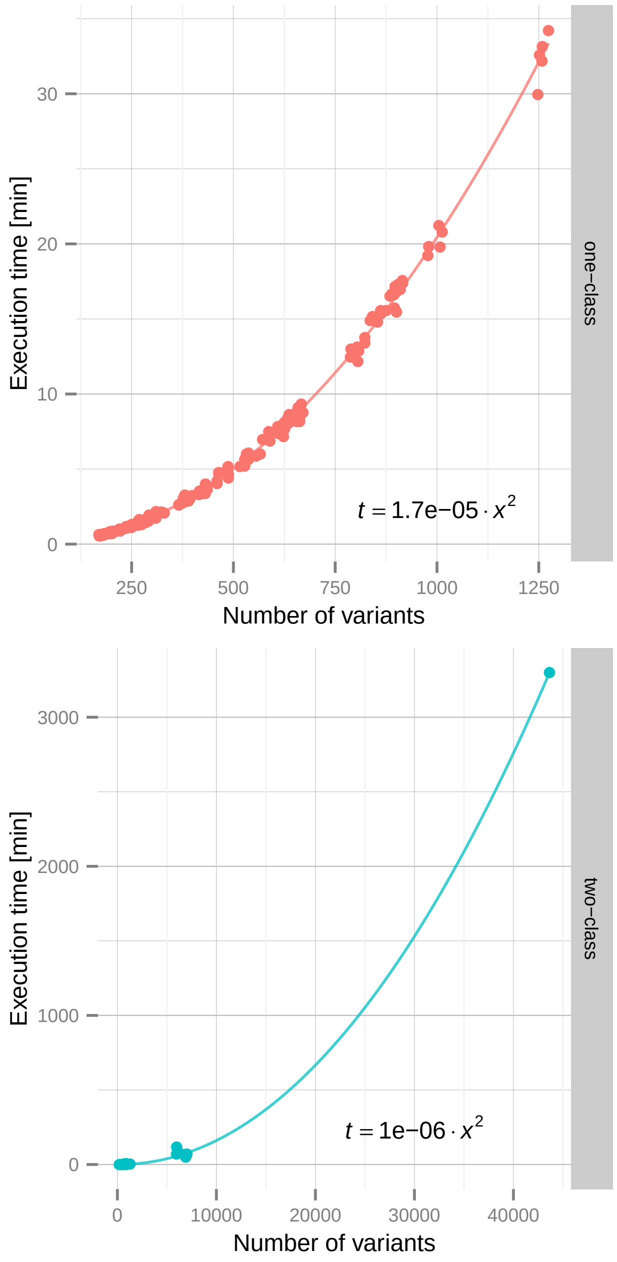

FIGURE S10. **Execution times of VariantMetaCaller.** Execution times (in minutes, using 1 CPU core) of VariantMetaCaller were plotted as the function of the number of variants. Quadratic functions fitted to the measurement points are shown as lines and the functions are also indicated. Top: one-class SVM, used for cases where negative training examples are not available (typically for smaller target regions), Bottom: two-class SVM, used for cases where both positive and negative training samples are available (typically for larger target regions)

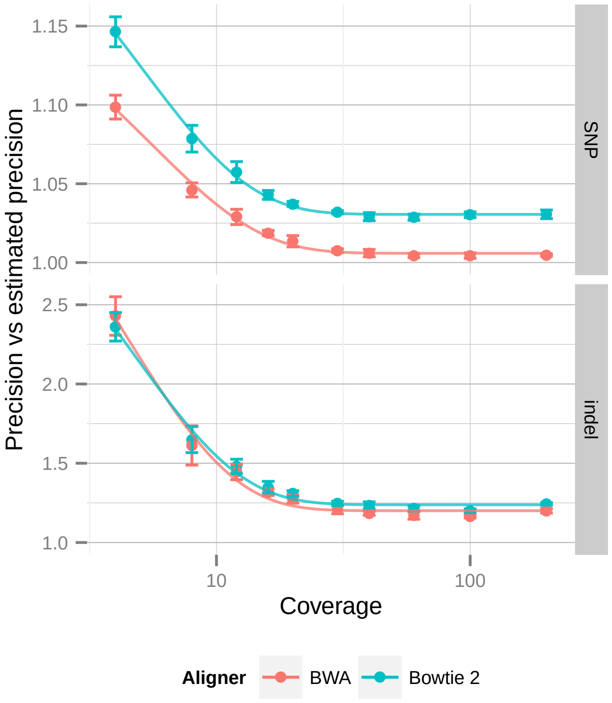

FIGURE S11. **Rate of true versus estimated precision in case of the simulated chromosome.**

Table S1. **AUPRC scores of VariantMetaCaller combining 3 or 4 individual variant callers.** The best performing methods are indicated with bold. AUPRC = area under the precision-recall curve, FB = FreeBayes, HC = HaplotypeCaller, ST = SAMtools, UG = UnifiedGenotyper, VMC = VariantMetaCaller

Type of variant	Coverage	BWA					Bowtie 2				
		HC UG FB	HC FB ST	HC UG ST	UG FB ST	HC UG FB ST	HC UG FB	HC FB ST	HC UG ST	UG FB ST	HC UG FB ST
SNPs	4	0.7046	0.7295	0.7272	0.7296	**0.7302**	0.6701	0.6981	0.6847	0.6989	**0.6992**
	8	0.8292	0.8448	0.8436	0.8443	**0.8456**	0.7922	0.8108	0.8042	0.8113	**0.8121**
	12	0.8781	0.8896	0.8892	0.8893	**0.8902**	0.8414	0.8557	0.8517	0.8559	**0.8565**
	16	0.9029	0.9110	0.9109	0.9107	**0.9115**	0.8677	0.8783	0.8763	0.8788	**0.8794**
	20	0.9178	0.9231	0.9232	0.9229	**0.9238**	0.8854	0.8925	0.8903	0.8930	**0.8935**
	30	0.9365	0.9390	0.9394	0.9388	**0.9398**	0.9065	0.9096	0.9078	0.9103	**0.9110**
	40	0.9442	0.9461	0.9467	0.9459	**0.9470**	0.9160	0.9185	0.9159	0.9195	**0.9199**
	60	0.9529	0.9540	0.9545	0.9539	**0.9546**	0.9264	0.9271	0.9236	0.9284	**0.9289**
	100	0.9596	0.9600	0.9607	0.9602	**0.9608**	0.9366	0.9357	0.9303	0.9376	**0.9379**
	200	0.9644	0.9641	0.9654	0.9648	**0.9654**	0.9443	0.9426	0.9359	0.9449	**0.9451**
Indels	4	0.5213	**0.5859**	0.5385	0.5579	0.5833	0.4923	**0.5407**	0.4996	0.5112	0.5392
	8	0.6864	**0.7228**	0.6898	0.6818	0.7181	0.6447	0.6718	0.6452	0.6349	**0.6737**
	12	0.7608	**0.7837**	0.7672	0.7440	0.7832	0.7121	0.7356	0.7233	0.7022	**0.7377**
	16	0.8008	0.8130	0.8011	0.7712	**0.8132**	0.7533	0.7661	0.7593	0.7288	**0.7680**
	20	0.8218	0.8321	0.8240	0.7921	**0.8332**	0.7724	0.7847	0.7821	0.7519	**0.7879**
	30	0.8558	0.8633	0.8592	0.8270	**0.8642**	0.8066	0.8131	0.8141	0.7831	**0.8179**
	40	0.8686	0.8700	0.8680	0.8405	**0.8737**	0.8239	0.8312	0.8320	0.8020	**0.8351**
	60	0.8806	0.8831	0.8814	0.8480	**0.8835**	0.8378	0.8390	0.8421	0.8091	**0.8440**
	100	0.8912	0.8919	0.8910	0.8596	**0.8937**	0.8508	0.8519	0.8537	0.8216	**0.8575**
	200	0.8994	0.8980	0.8998	0.8620	**0.9022**	0.8604	0.8587	0.8599	0.8250	**0.8638**

Table S2. **Sensitivity and precision of individual variant callers in case of the simulated chromosome.** The best performing methods are indicated with bold.

Type of variant	Aligner	Coverage	Sensitivity (95% CI)				Precision (95% CI)			
			HaplotypeCaller	UnifiedGenotyper	FreeBayes	SAMtools	HaplotypeCaller	UnifiedGenotyper	FreeBayes	SAMtools
SNPs	bwa	4	0.631 (0.611-0.651)	0.675 (0.658-0.691)	0.686 (0.665-0.708)	**0.724 (0.706-0.741)**	**0.997 (0.996-0.998)**	0.994 (0.993-0.995)	0.994 (0.993-0.996)	0.967 (0.963-0.971)
		8	0.792 (0.773-0.812)	0.807 (0.789-0.825)	0.819 (0.801-0.838)	**0.84 (0.822-0.858)**	**0.996 (0.995-0.997)**	0.992 (0.991-0.993)	0.993 (0.991-0.994)	0.984 (0.981-0.987)
		12	0.854 (0.832-0.876)	0.862 (0.842-0.883)	0.87 (0.849-0.891)	**0.885 (0.866-0.904)**	**0.997 (0.996-0.997)**	0.991 (0.991-0.992)	0.992 (0.992-0.993)	0.989 (0.987-0.99)
		16	0.886 (0.869-0.903)	0.892 (0.876-0.908)	0.894 (0.879-0.909)	**0.907 (0.892-0.922)**	**0.996 (0.995-0.997)**	0.99 (0.989-0.991)	0.992 (0.991-0.994)	0.989 (0.988-0.99)
		20	0.904 (0.89-0.919)	0.909 (0.895-0.923)	0.909 (0.896-0.922)	**0.919 (0.906-0.932)**	**0.996 (0.995-0.996)**	0.99 (0.989-0.99)	0.993 (0.992-0.993)	0.99 (0.989-0.991)
		30	0.927 (0.913-0.941)	0.93 (0.916-0.944)	0.927 (0.912-0.941)	**0.935 (0.922-0.948)**	**0.996 (0.995-0.997)**	0.988 (0.987-0.99)	0.992 (0.991-0.993)	0.989 (0.987-0.991)
		40	0.936 (0.926-0.947)	0.939 (0.929-0.95)	0.934 (0.922-0.946)	**0.942 (0.931-0.953)**	**0.995 (0.994-0.996)**	0.987 (0.985-0.989)	0.992 (0.991-0.993)	0.989 (0.987-0.99)
		60	0.946 (0.935-0.957)	**0.949 (0.938-0.96)**	0.941 (0.929-0.954)	**0.949 (0.938-0.96)**	**0.994 (0.993-0.995)**	0.986 (0.984-0.988)	0.992 (0.991-0.993)	0.989 (0.988-0.99)
		100	0.954 (0.945-0.963)	**0.956 (0.946-0.966)**	0.946 (0.934-0.958)	0.954 (0.945-0.963)	**0.994 (0.993-0.995)**	0.984 (0.982-0.985)	0.992 (0.992-0.993)	0.989 (0.987-0.99)
		200	0.959 (0.95-0.968)	**0.961 (0.952-0.971)**	0.95 (0.939-0.962)	0.955 (0.946-0.965)	**0.994 (0.993-0.995)**	0.983 (0.981-0.984)	0.992 (0.991-0.993)	0.99 (0.988-0.991)
	bowtie2	4	0.585 (0.567-0.602)	0.634 (0.619-0.648)	0.65 (0.633-0.668)	**0.68 (0.665-0.696)**	**0.997 (0.996-0.998)**	0.992 (0.99-0.994)	0.99 (0.988-0.992)	0.967 (0.965-0.97)
		8	0.736 (0.718-0.755)	0.765 (0.746-0.784)	0.779 (0.761-0.797)	**0.8 (0.783-0.818)**	**0.998 (0.997-0.999)**	0.989 (0.988-0.99)	0.991 (0.99-0.992)	0.985 (0.983-0.987)
		12	0.794 (0.773-0.815)	0.82 (0.801-0.839)	0.831 (0.811-0.852)	**0.848 (0.83-0.867)**	**0.998 (0.998-0.999)**	0.986 (0.984-0.988)	0.989 (0.988-0.99)	0.988 (0.986-0.99)
		16	0.826 (0.809-0.843)	0.85 (0.835-0.865)	0.857 (0.844-0.871)	**0.873 (0.859-0.887)**	**0.998 (0.998-0.999)**	0.984 (0.982-0.987)	0.989 (0.987-0.99)	0.988 (0.987-0.99)
		20	0.845 (0.832-0.859)	0.868 (0.854-0.882)	0.876 (0.862-0.889)	**0.887 (0.874-0.901)**	**0.998 (0.998-0.999)**	0.983 (0.98-0.986)	0.99 (0.988-0.991)	0.989 (0.988-0.99)
		30	0.867 (0.853-0.881)	0.89 (0.876-0.904)	0.897 (0.883-0.911)	**0.904 (0.891-0.917)**	**0.999 (0.998-0.999)**	0.982 (0.979-0.984)	0.989 (0.987-0.991)	0.989 (0.987-0.99)
		40	0.877 (0.866-0.888)	0.899 (0.888-0.91)	0.906 (0.895-0.918)	**0.912 (0.901-0.923)**	**0.999 (0.998-0.999)**	0.979 (0.976-0.981)	0.988 (0.987-0.989)	0.988 (0.987-0.989)
		60	0.888 (0.875-0.9)	0.91 (0.899-0.922)	0.916 (0.903-0.928)	**0.919 (0.907-0.931)**	**0.999 (0.999-0.999)**	0.976 (0.973-0.979)	0.987 (0.986-0.989)	0.988 (0.987-0.989)
		100	0.897 (0.887-0.906)	0.919 (0.909-0.93)	**0.924 (0.912-0.936)**	**0.924 (0.915-0.934)**	**0.999 (0.998-0.999)**	0.972 (0.967-0.977)	0.986 (0.983-0.988)	0.987 (0.987-0.988)
		200	0.904 (0.895-0.914)	0.927 (0.917-0.937)	**0.931 (0.919-0.943)**	0.926 (0.916-0.936)	**0.999 (0.998-0.999)**	0.969 (0.966-0.973)	0.986 (0.984-0.987)	0.987 (0.985-0.988)
Indels	bwa	4	**0.463 (0.442-0.484)**	0.311 (0.3-0.322)	0.45 (0.432-0.468)	0.439 (0.42-0.458)	0.912 (0.893-0.931)	**0.967 (0.96-0.974)**	0.951 (0.944-0.958)	0.847 (0.832-0.862)
		8	**0.653 (0.624-0.681)**	0.48 (0.465-0.495)	0.578 (0.557-0.6)	0.54 (0.528-0.553)	0.906 (0.894-0.919)	**0.949 (0.937-0.96)**	0.939 (0.927-0.95)	0.821 (0.799-0.842)
		12	**0.74 (0.722-0.758)**	0.601 (0.582-0.62)	0.638 (0.62-0.656)	0.593 (0.581-0.604)	0.904 (0.898-0.91)	**0.94 (0.935-0.946)**	0.93 (0.919-0.941)	0.8 (0.78-0.819)
		16	**0.783 (0.768-0.798)**	0.678 (0.662-0.694)	0.673 (0.653-0.692)	0.616 (0.607-0.625)	0.906 (0.897-0.916)	**0.935 (0.927-0.942)**	0.927 (0.924-0.93)	0.765 (0.75-0.779)
		20	**0.807 (0.791-0.823)**	0.717 (0.696-0.738)	0.696 (0.672-0.72)	0.643 (0.634-0.652)	0.906 (0.896-0.915)	**0.933 (0.928-0.937)**	0.924 (0.918-0.931)	0.742 (0.729-0.756)
		30	**0.843 (0.823-0.864)**	0.781 (0.764-0.798)	0.726 (0.701-0.75)	0.674 (0.661-0.688)	0.907 (0.896-0.918)	**0.924 (0.913-0.934)**	0.918 (0.913-0.924)	0.697 (0.68-0.715)
		40	**0.855 (0.837-0.873)**	0.802 (0.783-0.821)	0.739 (0.718-0.76)	0.686 (0.676-0.697)	0.908 (0.898-0.918)	**0.925 (0.92-0.931)**	0.919 (0.91-0.928)	0.679 (0.66-0.697)
		60	**0.869 (0.85-0.888)**	0.817 (0.799-0.834)	0.752 (0.725-0.779)	0.695 (0.679-0.711)	0.906 (0.895-0.918)	**0.926 (0.915-0.938)**	0.915 (0.91-0.921)	0.683 (0.675-0.69)
		100	**0.879 (0.861-0.898)**	0.835 (0.817-0.853)	0.768 (0.744-0.791)	0.694 (0.681-0.706)	0.907 (0.897-0.917)	**0.923 (0.915-0.932)**	0.912 (0.91-0.915)	0.669 (0.646-0.691)
		200	**0.888 (0.871-0.904)**	0.847 (0.827-0.866)	0.776 (0.751-0.8)	0.634 (0.619-0.648)	0.909 (0.897-0.92)	**0.923 (0.911-0.935)**	0.909 (0.905-0.912)	0.659 (0.643-0.675)
	bowtie2	4	**0.432 (0.409-0.455)**	0.3 (0.294-0.306)	0.418 (0.4-0.437)	0.398 (0.38-0.415)	0.944 (0.933-0.956)	**0.975 (0.967-0.983)**	0.95 (0.942-0.958)	0.831 (0.821-0.841)
		8	**0.606 (0.577-0.636)**	0.459 (0.439-0.479)	0.538 (0.519-0.556)	0.502 (0.489-0.514)	0.929 (0.922-0.936)	**0.952 (0.941-0.962)**	0.935 (0.926-0.944)	0.795 (0.776-0.813)
		12	**0.685 (0.668-0.702)**	0.57 (0.556-0.584)	0.594 (0.577-0.61)	0.563 (0.553-0.573)	0.921 (0.913-0.929)	**0.947 (0.938-0.956)**	0.93 (0.921-0.939)	0.765 (0.75-0.78)
		16	**0.729 (0.714-0.744)**	0.644 (0.628-0.66)	0.625 (0.61-0.641)	0.585 (0.576-0.595)	0.921 (0.911-0.932)	**0.938 (0.933-0.942)**	0.923 (0.917-0.929)	0.722 (0.709-0.736)
		20	**0.75 (0.735-0.765)**	0.681 (0.662-0.7)	0.647 (0.623-0.67)	0.612 (0.607-0.618)	0.919 (0.912-0.926)	**0.931 (0.924-0.937)**	0.921 (0.909-0.932)	0.703 (0.683-0.724)
		30	**0.783 (0.765-0.802)**	0.74 (0.724-0.757)	0.679 (0.657-0.701)	0.643 (0.635-0.65)	0.915 (0.907-0.923)	**0.921 (0.914-0.928)**	0.914 (0.903-0.926)	0.653 (0.633-0.673)
		40	**0.797 (0.778-0.817)**	0.761 (0.744-0.777)	0.695 (0.678-0.712)	0.659 (0.652-0.666)	0.914 (0.905-0.923)	**0.921 (0.913-0.93)**	0.911 (0.902-0.92)	0.633 (0.611-0.655)
		60	**0.811 (0.791-0.831)**	0.779 (0.76-0.799)	0.713 (0.69-0.736)	0.666 (0.655-0.677)	0.91 (0.901-0.919)	**0.918 (0.909-0.926)**	0.908 (0.903-0.913)	0.623 (0.614-0.633)
		100	**0.824 (0.806-0.843)**	0.793 (0.776-0.811)	0.729 (0.706-0.753)	0.664 (0.657-0.67)	0.906 (0.892-0.919)	**0.91 (0.902-0.918)**	0.904 (0.898-0.909)	0.601 (0.587-0.615)
		200	**0.833 (0.815-0.85)**	0.806 (0.788-0.823)	0.739 (0.719-0.758)	0.6 (0.584-0.616)	0.908 (0.897-0.92)	**0.905 (0.893-0.917)**	0.898 (0.894-0.903)	0.575 (0.554-0.595)

Table S3. **AUPRC scores and differences between AUPRC of VariantMetaCaller and each variant caller in case of the simulated chromosome.** AUPRC = area under the precision-recall curve, VMC = VariantMetaCaller

Type of variant	Aligner	Coverage	AUPRC of VMC	HaplotypeCaller			UnifiedGenotyper			freebayes			samtools		
				AUPRC	Difference of AUPRC	p-value[1]	AUPRC	Difference of AUPRC	p-value[1]	AUPRC	Difference of AUPRC	p-value[1]	AUPRC	Difference of AUPRC	p-value[1]
SNPs	bwa	4	0.730	0.622	0.109	6.11E-07	0.660	0.071	3.34E-06	0.380	0.350	1.33E-08	0.420	0.310	4.43E-09
		8	0.846	0.777	0.068	3.70E-06	0.786	0.060	5.35E-07	0.555	0.291	8.09E-08	0.578	0.268	1.10E-07
		12	0.890	0.837	0.054	1.73E-06	0.838	0.052	2.30E-06	0.673	0.217	3.90E-07	0.686	0.204	3.17E-07
		16	0.912	0.867	0.045	3.42E-07	0.864	0.048	1.01E-07	0.748	0.163	1.77E-07	0.757	0.155	1.80E-07
		20	0.924	0.884	0.039	7.64E-06	0.878	0.046	9.47E-07	0.800	0.123	4.54E-06	0.806	0.118	3.78E-06
		30	0.940	0.905	0.035	2.18E-08	0.887	0.053	1.53E-06	0.866	0.074	3.54E-06	0.867	0.073	7.38E-06
		40	0.947	0.911	0.036	1.26E-06	0.888	0.059	5.43E-07	0.890	0.057	2.13E-05	0.889	0.058	1.52E-05
		60	0.955	0.919	0.036	3.38E-07	0.881	0.074	1.54E-06	0.912	0.042	2.58E-05	0.910	0.045	2.18E-05
		100	0.961	0.924	0.037	6.80E-07	0.865	0.096	3.10E-07	0.928	0.032	1.02E-05	0.924	0.036	1.91E-05
		200	0.965	0.926	0.040	8.30E-07	0.727	0.239	2.74E-06	0.940	0.026	2.21E-05	0.934	0.031	1.40E-05
	bowtie2	4	0.699	0.561	0.138	9.65E-08	0.584	0.115	7.77E-08	0.345	0.355	3.42E-09	0.375	0.324	1.81E-09
		8	0.812	0.698	0.114	4.02E-07	0.695	0.117	3.92E-08	0.511	0.301	8.71E-08	0.519	0.293	7.42E-08
		12	0.856	0.749	0.108	3.18E-08	0.741	0.115	3.32E-09	0.625	0.231	1.16E-07	0.622	0.235	7.54E-08
		16	0.879	0.775	0.105	1.44E-09	0.764	0.116	8.17E-11	0.701	0.179	1.76E-07	0.696	0.184	7.49E-08
		20	0.894	0.789	0.105	5.04E-08	0.776	0.117	1.70E-08	0.753	0.141	2.18E-06	0.746	0.148	3.56E-07
		30	0.911	0.805	0.106	9.34E-09	0.786	0.125	2.81E-08	0.824	0.087	3.44E-06	0.814	0.097	1.12E-06
		40	0.920	0.812	0.108	5.20E-08	0.789	0.131	1.58E-08	0.850	0.070	1.22E-05	0.837	0.082	4.68E-06
		60	0.929	0.819	0.110	2.13E-08	0.787	0.142	3.04E-08	0.880	0.049	1.02E-05	0.862	0.067	1.85E-06
		100	0.938	0.823	0.115	1.22E-07	0.776	0.162	5.70E-08	0.901	0.037	9.14E-06	0.878	0.060	5.10E-06
		200	0.945	0.825	0.121	1.09E-08	0.647	0.298	7.49E-07	0.917	0.028	1.80E-05	0.889	0.056	4.89E-06
Indels	bwa	4	0.583	0.425	0.158	4.90E-06	0.294	0.289	3.20E-07	0.205	0.378	4.06E-07	0.220	0.364	4.70E-08
		8	0.718	0.592	0.125	1.90E-06	0.436	0.282	4.72E-06	0.332	0.386	2.27E-06	0.297	0.421	1.99E-06
		12	0.783	0.672	0.111	4.25E-05	0.538	0.245	4.88E-06	0.421	0.363	2.81E-06	0.338	0.445	5.31E-07
		16	0.813	0.709	0.104	1.67E-05	0.600	0.213	7.42E-07	0.477	0.336	5.59E-07	0.349	0.464	7.69E-08
		20	0.834	0.732	0.102	5.86E-05	0.631	0.204	1.68E-07	0.523	0.311	1.59E-07	0.359	0.476	5.70E-07
		30	0.865	0.763	0.102	1.16E-05	0.681	0.183	3.19E-06	0.592	0.273	3.78E-06	0.373	0.492	4.18E-07
		40	0.873	0.775	0.098	2.33E-05	0.700	0.173	2.11E-08	0.622	0.250	1.90E-06	0.368	0.504	4.83E-07
		60	0.884	0.790	0.093	2.85E-05	0.714	0.169	8.19E-07	0.652	0.232	5.74E-06	0.385	0.498	2.89E-07
		100	0.894	0.798	0.095	7.01E-05	0.728	0.166	8.92E-06	0.676	0.217	2.14E-07	0.382	0.512	6.35E-07
		200	0.902	0.806	0.096	2.59E-05	0.735	0.167	5.03E-06	0.689	0.213	7.70E-07	0.351	0.551	2.44E-07
	bowtie2	4	0.539	0.416	0.123	1.16E-06	0.292	0.247	4.18E-06	0.187	0.352	6.56E-07	0.189	0.350	1.52E-08
		8	0.674	0.574	0.100	1.35E-06	0.434	0.240	8.81E-06	0.307	0.367	1.00E-06	0.259	0.415	1.45E-06
		12	0.738	0.645	0.093	9.01E-06	0.535	0.203	9.24E-06	0.395	0.343	2.03E-06	0.300	0.438	9.33E-08
		16	0.768	0.684	0.084	2.02E-05	0.596	0.172	3.77E-07	0.445	0.323	2.03E-06	0.316	0.452	3.59E-07
		20	0.788	0.703	0.085	3.41E-07	0.628	0.160	7.89E-07	0.489	0.299	1.70E-06	0.326	0.461	2.22E-07
		30	0.817	0.731	0.087	2.51E-06	0.672	0.146	5.83E-06	0.555	0.262	4.83E-06	0.341	0.476	8.40E-07
		40	0.835	0.742	0.093	9.45E-07	0.692	0.143	2.04E-05	0.580	0.255	1.03E-07	0.336	0.499	7.32E-07
		60	0.844	0.755	0.089	1.71E-05	0.706	0.138	3.07E-06	0.611	0.233	3.33E-07	0.348	0.496	1.92E-07
		100	0.858	0.765	0.093	1.51E-05	0.714	0.144	1.60E-05	0.637	0.221	9.86E-07	0.345	0.513	8.73E-07
		200	0.864	0.769	0.095	6.82E-06	0.718	0.146	5.34E-06	0.651	0.213	4.62E-07	0.298	0.566	4.13E-07

[1] P-value of paired, two-tailed t-test

Table S4. **Effects of the differences between BWA and Bowtie 2 alignments on the sensitivity and precision of the individual variant callers in case of the simulated chromosome**

Type of variant	Variant caller	Sensitivity			Precision		
		Mean difference	95% CI	p-value[1]	Mean difference	95% CI	p-value[1]
SNPs	HaplotypeCaller	0.057	0.056-0.058	3.01E-57	-0.003	-0.003--0.002	2.19E-18
	UnifiedGenotyper	0.04	0.039-0.041	6.71E-57	0.007	0.006-0.008	1.40E-17
	FreeBayes	0.031	0.029-0.033	1.49E-33	0.004	0.003-0.005	2.29E-20
	SAMtools	0.034	0.032-0.035	7.45E-44	0.001	0-0.001	2.01E-04
Indels	HaplotypeCaller	0.053	0.05-0.055	5.55E-40	-0.012	-0.015--0.008	1.63E-08
	UnifiedGenotyper	0.034	0.03-0.037	3.30E-26	0.003	0-0.006	8.25E-02
	FreeBayes	0.042	0.04-0.044	2.30E-38	0.005	0.003-0.007	4.16E-07
	SAMtools	0.032	0.03-0.034	1.06E-36	0.046	0.04-0.052	8.89E-21

[1] P-value of paired, two-tailed t-test

Table S5. **Effects of the differences between BWA and Bowtie 2 alignments on the maximal sensitivity of VariantMetaCaller in case of the simulated chromosome**

Type of variant	Coverage	Maximum sensitivity of VariantMetaCaller				
		Alignment		Mean difference	95% CI	p-value[1]
		bwa	bowtie2			
SNP	4	0.731	0.700	0.031	0.027-0.034	0.000015
	8	0.846	0.813	0.033	0.032-0.035	0.000001
	12	0.891	0.857	0.034	0.031-0.036	0.000005
	16	0.912	0.880	0.032	0.03-0.034	0.000001
	20	0.924	0.894	0.030	0.028-0.032	0.000001
	30	0.940	0.911	0.029	0.027-0.031	0.000002
	40	0.947	0.920	0.027	0.025-0.029	0.000001
	60	0.955	0.929	0.026	0.023-0.028	0.000007
	100	0.961	0.939	0.023	0.021-0.024	0.000001
	200	0.966	0.946	0.020	0.019-0.021	0.000001
Indel	4	0.605	0.555	0.050	0.041-0.058	0.000094
	8	0.741	0.690	0.051	0.046-0.055	0.000008
	12	0.803	0.754	0.049	0.044-0.054	0.000012
	16	0.830	0.782	0.047	0.038-0.057	0.000162
	20	0.850	0.804	0.047	0.04-0.053	0.000048
	30	0.880	0.831	0.048	0.044-0.053	0.000009
	40	0.888	0.849	0.039	0.035-0.042	0.000009
	60	0.899	0.858	0.041	0.033-0.048	0.000102
	100	0.908	0.872	0.036	0.032-0.04	0.000016
	200	0.917	0.880	0.037	0.032-0.042	0.000038

[1] P-value of paired, two-tailed t-test

TABLE S6. **Number of different types of variants in the simulated data of different target sizes**

Size of target region	Average number of SNPs (s.d.)	Average number of indels (s.d.)	Average number of polymorphic SNPs per sample (s.d.)	Average number of polymorphic indels per sample (s.d.)
100 Kb	450 (73.8)	60 (22.0)	98 (17.1)	14 (4.9)
200 Kb	814 (159.3)	111 (30.6)	181 (44.3)	26 (7.1)
300 Kb	1185 (192.8)	160 (31.7)	262 (55.4)	40 (10.5)
500 Kb	1976 (238.5)	267 (30.8)	436 (53.2)	67 (9.3)
