# Supplementary material for: VariantMetaCaller: automated fusion of variant calling pipelines for quantitative, precision-based filtering
Source: BMC Genomics. 2015 Oct 28;16:875. doi: 10.1186/s12864-015-2050-y (PMC4625715; doi:10.1186/s12864-015-2050-y)
Supplement: Additional file 2 — Variant annotations used as features for SVMs. The full listing and short description of the variant annotations used as features for Support Vector Machines. [file 12864_2015_2050_MOESM2_ESM.pdf]

Annotation (Feature)	Description	Used for SNPs				Used for Indels				Type						
		GATK HaplotypeCaller	GATK UnifiedGenotyper	freebayes	samtools	GATK HaplotypeCaller	GATK UnifiedGenotyper	freebayes	samtools	Mapping quality	Base quality	Allele balance	Strand bias	Sequence complexity	Genotype evidence	Other quality
AB	Allele balance at heterozygous sites: a number between 0 and 1 representing the ratio of reads showing the reference allele to all reads, considering only reads from individuals called as heterozygous			+				+				+				
ABHet	Allele Balance for heterozygous calls (ref/(ref+alt))		+									+				
ABHom	Allele Balance for homozygous calls (A/(A+O)) where A is the allele (ref or alt) and O is anything other		+									+				
ABP	Allele balance probability at heterozygous sites: Phred-scaled upper-bounds estimate of the probability of observing the deviation between ABR and ABA given E(ABR/ABA) ~ 0.5, derived using Hoeffding's inequality			+				+				+				
BaseQRankSum	Z-score from Wilcoxon rank sum test of Alt Vs. Ref base qualities	+	+			+	+				+					
BasesToClosestVariant	Number of bases to the closest variant	+	+	+	+	+	+	+	+							+
BQB	Mann-Whitney U test of Base Quality Bias				+						+					
ClippingRankSum	Z-score From Wilcoxon rank sum test of Alt vs. Ref number of hard clipped bases	+				+										+
DP	Approximate read depth	+	+	+	+	+	+	+	+							
EntropyCenter_15	Shannon entropy of the reference sequence of size 15 around the variant	+	+	+	+	+	+	+	+					+		
EntropyCenter_7	Shannon entropy of the reference sequence of size 7 around the variant	+	+	+	+	+	+	+	+					+		
EntropyLeft_15	Shannon entropy of the reference sequence of size 15 to the left of the variant	+	+	+	+	+	+	+	+					+		
EntropyLeft_7	Shannon entropy of the reference sequence of size 7 to the left of the variant	+	+	+	+	+	+	+	+					+		
EntropyRight_15	Shannon entropy of the reference sequence of size 15 to the right of the variant	+	+	+	+	+	+	+	+					+		
EntropyRight_7	Shannon entropy of the reference sequence of size 7 to the right of the variant	+	+	+	+	+	+	+	+					+		
FS	Phred-scaled p-value using Fisher's exact test to detect strand bias	+	+			+	+						+			
GC	GC content around the variant		+				+							+		
GenotypeEntropyMean	Mean of the entropies of the distributions defined by genotype likelihoods across all samples	+	+	+	+	+	+	+	+						+	
GenotypeEntropySD	Standard deviation of the entropies of the distributions defined by genotype likelihoods across all samples	+	+	+	+	+	+	+	+						+	
HaplotypeScore	Consistency of the site with at most two segregating haplotypes		+													+
HOB	Bias in the number of HOMs number				+				+							

HRun	Largest Contiguous Homopolymer Run of Variant Allele In Either Direction		+				+							+		
ICB	Inbreeding Coefficient Binomial test				+				+						+	
IDV	Maximum number of reads supporting an indel								+						+	
IMF	Maximum fraction of reads supporting an indel								+						+	
InbreedingCoeff	Inbreeding coefficient as estimated from the genotype likelihoods per-sample when compared against the Hardy-Weinberg expectation	+	+			+	+								+	
LikelihoodRankSum	Z-score from Wilcoxon rank sum test of Alt Vs. Ref haplotype likelihoods	+				+	+									+
MQ	RMS Mapping Quality	+	+		+	+	+		+	+						
MQB	Mann-Whitney U test of Mapping Quality Bias				+					+						
MQM	Mean mapping quality of observed alternate alleles			+				+		+						
MQMR	Mean mapping quality of observed reference alleles			+				+		+						
MQRankSum	Z-score From Wilcoxon rank sum test of Alt vs. Ref read mapping qualities	+	+			+	+			+						
MQSB	Mann-Whitney U test of Mapping Quality vs Strand Bias				+				+							+
ODDS	The log odds ratio of the best genotype combination to the second-best			+				+							+	
QD	Variant Confidence/Quality by Depth	+	+			+	+									+
QUAL	Phred-scaled quality score estimating the probability that there is a polymorphism at the loci	+	+	+	+	+	+	+	+							+
SAP	Strand balance probability for the alternate allele: Phred-scaled upper-bounds estimate of the probability of observing the deviation between number of alternate observations on the forward strand (SAF) and number of alternate observations on the forward strand (SAR) given E(SAF/SAR) ~ 0.5, derived using Hoeffding's inequality			+				+					+			
SGB[1]	Segregation based metric				+				+			(+)			(+)	
SOR	Symmetric Odds Ratio of 2x2 contingency table to detect strand bias	+	+			+	+						+			
SRP	Strand balance probability for the reference allele: Phred-scaled upper-bounds estimate of the probability of observing the deviation between number of reference observations on the forward strand (SRF) and number of reference observations on the reverse strand (SRR) given E(SRF/SRR) ~ 0.5, derived using Hoeffding's inequality			+				+					+			

Additional references

[1] http://samtools.github.io/bcftools/rd-SegBias.pdf
